# Bioactive Compounds from *Dodonaea viscosa* Flowers: Potent Antibacterial and Antiproliferative Effects in Breast Cancer Cells

**DOI:** 10.3390/molecules30112274

**Published:** 2025-05-22

**Authors:** Achara Raksat, Daniel Yee, Young Jin Gi, Supakit Wongwiwatthananukit, Leng Kar Chang, Kumu Piilani Kaawaloa, Marisa M. Wall, Jangsoon Lee, Leng Chee Chang

**Affiliations:** 1 Department of Pharmaceutical Sciences, The Daniel K. Inouye College of Pharmacy, University of Hawai‘i at Hilo, Hilo, HI 96720, USA; achara@hawaii.edu (A.R.); yeeds@hawaii.edu (D.Y.); 2 Preclinical Core, Cancer Biology Program, University of Hawai‘i Cancer Center, Honolulu, HI 96813, USA; ygi@cc.hawaii.edu; 3 Department of Pharmacy Practice, The Daniel K. Inouye College of Pharmacy, University of Hawai‘i at Hilo, Hilo, HI 96720, USA; supakit@hawaii.edu; 4 Georgia Institute of Science and Research Incorporation, Duluth, GA 30096, USA; lchang@gaisr.org; 5 Hawaiian Language/Performing Arts, Kamehameha Schools, Hawai‘i Campus, Kea’au, HI 96749, USA; pikaawal@ksbe.edu; 6 Daniel K. Inouye U.S. Pacific Basin Agricultural Research Center, Hilo, HI 96720, USA; marisa.wall@usda.gov

**Keywords:** *Dodonaea viscosa*, Hawaiian plant, antibacterial activity, flavonoids, breast cancer, antiproliferative effect, bioactive natural compounds

## Abstract

*Dodonaea viscosa* (Sapindaceae), a Hawaiian local medicinal plant, has been traditionally used to treat rashes and skin diseases. The study aimed to discover and characterize bioactive compounds from *D. viscosa* flowers extract with antimicrobial and antitumor properties. Thirteen compounds were isolated from the methanol extract of *D. viscosa* flowers, and their structures were characterized using spectroscopy data, comparing their NMR spectroscopic profiles with previously reported data. Subsequent antibacterial assays revealed that one particular compound, **12**, exhibited significant antimicrobial activity against Gram-positive bacteria. Notably, it demonstrated a minimum inhibitory concentration (MIC) of 2 μg/mL, indicating its potent antibacterial potential. In addition to antimicrobial properties, the isolated compounds demonstrated dose-dependent antiproliferative effects in breast cancer cell lines. Notably, inflammatory breast cancer (IBC) cell lines, a highly aggressive subtype, were more sensitive to compound **6**, with IC_50_ values of 4.22 μM (BCX-010), 6.74 μM (SUM190), and 7.73 (SUM149), compared to non-IBC cell line. These findings highlight the dual antibacterial and anticancer potential of compounds from *D. viscosa*, emphasizing their promise as candidates for therapeutic development.

## 1. Introduction

The integration of traditional Hawaiian medicine and modern scientific research underscores the considerable therapeutic potential of *Dodonaea viscosa*, commonly known as ‘A‘ali’i. For centuries, Native Hawaiians have harnessed the resilience of this plant to treat various ailments, including toothaches, fever, and skin irritations [1]. This historical application suggests the presence of bioactive compounds with analgesic, antipyretic, and anti-inflammatory properties. Contemporary phytochemical studies have revealed a diverse array of constituents in *D. viscosa*, including alkaloids, flavonoids, glycosides, phenols, terpenoids, and steroids [2,3,4,5,6,7]. These varied chemical compounds are recognized for their extensive biological activities, positioning *D. viscosa* as a promising candidate in the fight against significant health challenges such as antimicrobial resistance and cancer.

The escalating threat of antibiotic-resistant bacteria, particularly methicillin-resistant *Staphylococcus aureus* (MRSA), is a critical public health crisis that requires urgent global attention, including within the Hawaiian Islands [8,9]. The disproportionately high rates of community-acquired MRSA infections among Native Hawaiian, Pacific Islander, and pediatric populations in Hawai‘i underscore the urgent need for novel antimicrobial agents [9,10,11]. Intriguingly, a specific clerodane-type diterpenoid isolated from *D. viscosa*, 6β-hydroxy-15,16-epoxy-5β,8β,9β,10α-cleroda-3,13(16),14-trien-18-oic acid, has demonstrated bacteriostatic activity against certain bacterial strains [12]. This discovery suggests that *D. viscosa* may serve as a valuable natural reservoir of antibacterial compounds capable of combating multidrug-resistant microorganisms. Further research into isolating and characterizing other antibacterial constituents from this plant could lead to the development of innovative therapeutic strategies against persistent bacterial infections.

In parallel, breast cancer, the most prevalent malignancy in women worldwide, remains a major clinical challenge due to its heterogeneity and resistance to therapy [13,14]. Metastatic breast cancer (mBC), particularly HER2-negative subtypes such as triple-negative breast cancer (TNBC), inflammatory breast cancer (IBC), and hormone receptor-positive (HR+) breast cancer, is associated with poor prognoses and limited treatment options. TNBC, characterized by the absence of hormone receptors and HER2 expression, exhibits high recurrence rates, metastasis, and poor overall survival [15,16]. Similarly, resistance to endocrine therapy in HR+ breast cancer [17] and the aggressive nature of IBC underscore the urgent need for innovative therapeutic strategies [18].

Preliminary investigations have indicated that *D. viscosa* flower extracts possess anti-proliferative effects in breast cancer cell lines. This highlights their potential as dual-action agents, possessing both antibacterial and anticancer properties [19,20,21]. The current study aimed to isolate and characterize bioactive compounds from the flowers of *D. viscosa*, evaluate their antimicrobial efficacy, and investigate their potential to inhibit the proliferation of breast cancer cells. These findings could pave the way for developing novel therapeutics to address both infectious disease and cancer challenges.

## 2. Results and Discussion

### 2.1. Isolated Compounds from D. viscosa Flowers

**Figure 1:**
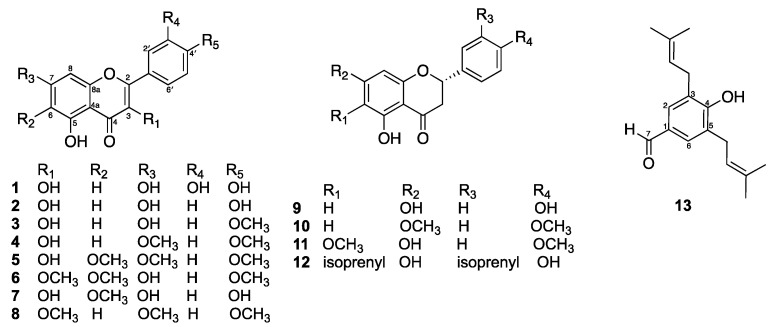
Structures of compounds **1**–**13** from *D. viscosa* flowers extract.

The MeOH extract of fresh flowers of *D. viscosa* was fractionated by silica gel and Sephadex LH-20 column chromatography to afford thirteen compounds identified as quercetin (**1**) [22], kaempferol (**2**) [22], kaempferide (**3**) [23], 3,5-dihydroxy-7,4′-dimethoxyflavone (**4**) [24], mikanin (**5**) [25], santin (**6**) [26], 6-methoxykaempferol (**7**) [27], 5-hydroxy-3,7,4′-trimethoxyflavone (**8**) [28], naringenin (**9**) [29], 5-hydroxy-7,4′-dimethoxyflavone (**10**) [30], 2,3-dihydro-5,7-dihydroxy-6-methoxy-2-(4-methoxyphenyl)-4H-1-benzopyran-4-one (**11**) [31], macarangaflavanone B (**12**) [32], and 3,5-diprenyl-4-hydroxybenzaldehyde (**13**) [33] (Figure 1). The structures were elucidated using spectroscopic data, including NMR results and literature comparisons (can be found in Supplementary Materials).

Compounds **1**–**8** were obtained as yellow solids. The ^1^H NMR spectrum of compound **1** displayed the presence of a set of hydrogen-bonded hydroxy proton at *δ*_H_ 12.18 (1H, s, 6-OH) and a trisubstituted benzene ring at *δ*_H_ 7.80 (1H, d, *J* = 2.0 Hz, H-2′), 7.68 (1H, dd, *J* = 8.4, 2.0 Hz, H-6′), and 6.97 (1H, d, *J* = 8.4 Hz, H-5′). A pair of meta-coupled doublets observed at *δ*_H_ 6.25 (1H, d, *J* = 2.0 Hz) and 6.52 (1H, d, *J* = 2.0 Hz) were assigned as H-6 and H-8, respectively. Therefore, structure **1** has a flavonol skeleton, which is a type of flavonoid and was identified as quercetin (**1**) [22]. The ^1^H NMR spectrum of compound **2**, an analog of compound **1**. Notably, compound **2** exhibited a pair of para-substituted benzene rings, with signals at *δ*_H_ 8.08 (2H, d, *J* = 8.9 Hz, H-2′ and H-6′) and 6.91 (2H, d, *J* = 8.9 Hz, H-3′ and H-5′). A comparison of the NMR data of compound **2** with those of kaempferol [22] confirmed its structure. In addition, the ^1^H NMR spectra of compounds **3**–**8** showed structural similarities to compound **2**. Compound **3** displayed a methoxy group at *δ*_H_ 3.89 (3H, s), situated at C-4′. In contrast, compound **4** had two methoxy groups at *δ*_H_ 3.88 (6H, s), located at C-7 and C-4′. Furthermore, compound **5** contained three methoxy groups at *δ*_H_ 3.97 (3H, s), 3.93 (3H, s), and 3.89 (3H, s), positioned at C-7, C-6, and C-4′, respectively. Compound **6** displayed three methoxy groups at *δ*_H_ 4.07 (3H, s), 3.92 (3H, s), and 3.88 (3H, s), situated at C-6, C-4′, and C-3, respectively. Compound **7** had one methoxy group at *δ*_H_ 3.90 (3H, s), located at C-6. Compound **8** contained three methoxy groups, which were located as C-3, C-7, and C-4′ at *δ*_H_ 3.88 (3H, s), 3.92 (3H, s), and 3.90 (3H, s), respectively. Based on the spectral data and a comparison with literature [23,24,25,26,27,28], compounds **3**–**8** were identified as kaempferide (**3**), 3,5-dihydroxy-7,4′-dimethoxyflavone (**4**), mikanin (**5**), santin (**6**), 6-methoxykaempferol (**7**), and 5-hydroxy-3,7,4′-trimethoxyflavone (**8**).

Compounds **9**–**12** were obtained as pale-yellow solids. The ^1^H NMR spectroscopic data of compound **9** displayed characteristic resonances for a ABX spin system of a flavanone at *δ*_H_ 5.30 (1H, dd, *J* = 13.0, 3.0 Hz, H-2), 3.08 (1H, dd, *J* = 16.8, 13.0 Hz, H-3b) and 2.65 (1H, dd, *J* = 16.8, 3.0 Hz, H-3a), and a para-substituted benzene ring at *δ*_H_ 7.29 (2H, d, *J* = 8.5 Hz, H-2′ and H-6′) and 6.82 (2H, d, *J* = 8.5 Hz, H-3′ and H-5′). A pair of meta-coupled doublets observed at *δ*_H_ 5.89 (1H, d, *J* = 2.0 Hz) and 5.88 (1H, d, *J* = 2.0 Hz) were assigned as H-6 and H-8, respectively. From the above and literature data, compound **9** was identified as naringenin (**9**) [29]. The ^1^H NMR data of compound **10** were similar to those of **9**, except that the two hydroxy groups of compound **9** at C-7 and C-4′ were replaced by methoxy groups at *δ*_H_ 3.86 (3H, s) and 3.83 (3H, s), respectively. A comparison of the NMR data of compound **10** with those of 5-hydroxy-7,4′-dimethoxyflavone [30] confirmed its structure. The ^1^H NMR data of compound **11** indicate a structural similarity to compound **10**. However, a pair of meta-coupled doublets observed at *δ*_H_ 6.06 (1H, d, *J* = 2.0 Hz, H-6) and 6.09 (1H, d, *J* = 2.0 Hz, H-8) in compound **10** disappeared. In compound **10**, the proton at C-6 was replaced by a methoxy group that appeared at *δ*_H_ 3.83 (3H, s). Additionally, the methoxy group at C-7, which was noted at *δ*_H_ 3.86 (3H, s) in compound **10**, was substituted with a hydroxy group in compound **11**. Additionally, a singlet resonance at *δ*_H_ 6.11 (1H, s) was assigned to H-8. The identity of compound **11** was established by comparison of its ^1^H NMR data with those of 2,3-dihydro-5,7-dihydroxy-6-methoxy-2-(4-methoxyphenyl)-4H-1-benzopyran-4-one [31]. The ^1^H NMR data of compound **12** were similar to those of **11**, except that the methoxy group at C-6 and the proton at C-3′ in compound **11** were substituted by isoprenyl units, the first unit at *δ*_H_ 5.19 (1H, t, *J* = 7.2 Hz), 3.21 (2H, d, *J* = 7.2 Hz), 1.75 (3H, s), and 1.70 (3H, s), and the second unit located at C-3′ [*δ*_H_ 5.33 (1H, t, *J* = 7.2 Hz), 3.31 (2H, d, *J* = 7.2 Hz), 1.72 (3H, s), and 1.63 (3H, s)]. Compound **12** was identified as macarangaflavanone B by comparing its ^1^H NMR data with the existing literature [32].

Compound **13** was obtained as a white solid. The ^1^H NMR spectrum exhibited only one aromatic singlet at *δ*_H_ 7.56 (2H, s, H-2, and H-6), suggesting a specific arrangement due to the molecule’s symmetry. Additionally, a singlet at *δ*_H_ 9.82 (1H, s) indicated the presence of an adjacent aldehyde group located at C-1. This definitively identified the isoprenyl substituents at C-3 and C-5, with resonances observed at *δ*_H_ 5.34 (2H, t, *J* = 7.2 Hz), *δ*_H_ 3.43 (4H, d, *J* = 7.2 Hz), *δ*_H_ 1.82 (6H, s), and *δ*_H_ 1.80 (6H, s). Additionally, a singlet resonance at *δ*_H_ 6.07 (1H, s) was assigned to the hydroxy group at C-4. From the above data, compound **13** has a hydroxybenzaldehyde derivative and was identified from comparison with literature data as 3,5-diprenyl-4-hydroxybenzaldehyde [33].

The crude extract of *D. viscosa* has demonstrated significant biological activity, exhibiting properties such as antioxidant, antimicrobial, antidiabetic, gastroprotective, and hepatoprotective effects, as evidenced by both in vitro and in vivo studies [34,35]. Notably, the methanol extract from flowers of *D. viscosa* inhibited the growth of *Mycobacterium tuberculosis* (TB) in vitro. TB is a serious infectious disease that primarily affects the lungs and is transmitted through airborne droplets. The inhalation of even a small number of these droplets can lead to infection [36].

### 2.2. Antibacterial Activity

This investigation examined several bacterial strains, including nine Gram-positive (MRSA USA-300, MSSA LUU7, MSSA 8325-4, *S. aureus* 8384, MSSA ONE6, MSSA RI27, MSSA LUE1, MRSA N315 and MSSA Newman). In addition, three Gram-negative bacteria were tested: *Escherichia coli* (9637), *Klebsiella pneumoniae*, and *Pseudomonas aeruginosa*. The minimum inhibitory concentrations (MICs) for the crude extract, each compound, and positive controls (vancomycin and gentamycin) were assessed. Vancomycin, acting as a positive control for Gram-positive strains, displayed MICs ranging from 0.5 to 1 µg/mL for all MRSA and MSSA strains tested. Gentamycin exhibited MICs between 0.25 and 1 µg/mL against the Gram-negative bacteria. The *D. viscosa* flowers methanol extract showed weak antibacterial activity against all Gram-positive and Gram-negative tested strains (MIC 320–1280 μg/mL). Compound **12** exhibited potent activity against Gram-positive bacteria, with MIC values of 2 μg/mL, and showed moderate activity against Gram-negative bacteria with MIC values of 128 μg/mL. The remaining compounds displayed moderate antibacterial activity (MIC 64–128 μg/mL) or no activity against all tested strains, as summarized in Table 1. The literature indicates that three isolated flavonoids—kaempferol (**2**), kaempferide (**3**), and santin (**6**)—were found to inhibit the growth of *Staphylococcus aureus*, with MIC values of 63, 250, and 63 mg/mL, respectively. For *E. coli*, the MIC values were 16, 125, and 63 mg/mL; and for *P. aeruginosa,* the values were 63, 250, and 125 mg/mL, respectively [37].

In our investigation, quercetin (**1**) showed weak antibacterial activity against all tested strains, with MIC values of 128 µg/mL. Kaempferol (**2**) inhibited the growth of all Gram-positive strains, including MRSA and MSSA, with MIC values of 64 µg/mL, and also weak activity against all Gram-negative strains, with MICs of 128 µg/mL. In contrast, kaempferide (**3**) was inactive against these strains. The presence of hydroxyl groups on flavonoids positively influences their antibacterial effectiveness, while methoxy substitutions tend to reduce activity against Gram-positive bacteria [38,39]. For example, kaempferol (**2**) contains hydroxyl groups at the C-4′ position, whereas kaempferide (**3**) has a methoxy substitution at the same position, which renders it inactive.

Among the subclass of flavonoids, compounds **9**–**12** (Figure 1) are categorized as flavanones. Notably, macarangaflavanone B (**12**) is the only flavanone that contains two isoprenyl units: one at the C-6 position in the A ring and another at the C-3 position in the B ring. These isoprenyl units may enhance antibacterial activity by improving membrane penetration for lipophilic groups (compound **12** exhibited potent activity against Gram-positive bacteria, with MIC values of 2 μg/mL, and showed weak activity against Gram-negative bacteria with MIC values of 128 μg/mL), whereas compounds **9**–**11** were inactive. In parallel, the literature indicated macarangaflavanone B (**12**) was previously isolated from the leaves of *Macaranga pleiostemona*, a shrub that is endemic to New Guinea. It exhibited significant antibacterial activity against Gram-negative bacteria *E. coli* and *Micrococcus luteus*, with MIC values of 0.5 μg as determined by thin-layer chromatography (TLC) using the Bioautographic method. In comparison, the MIC value for chloramphenicol was 0.05 μg [40]. These findings suggest that macarangaflavanone B (**12**) may have potential as a lead compound for further evaluation and development as an antibacterial agent.

### 2.3. Compound **6** (Santin) from D. viscosa Flowers Inhibits Breast Cancer Cell Growth via G2-M Cell Cycle Arrest

**Figure 2:**
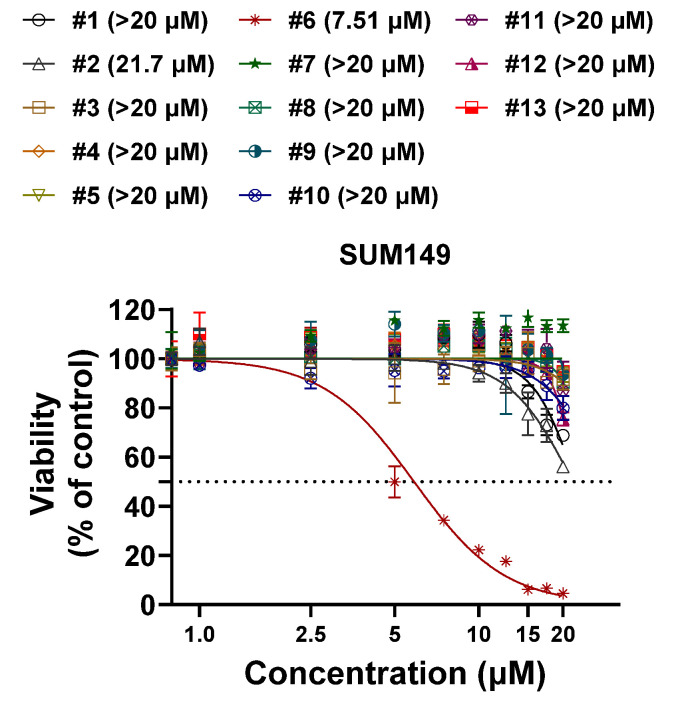
Compound **6** has the most effective antiproliferative effect compared to other compounds in SUM149 breast cancer cell lines. Sulforhodamine B assay was conducted for 5 days of incubation.

**Figure 3:**
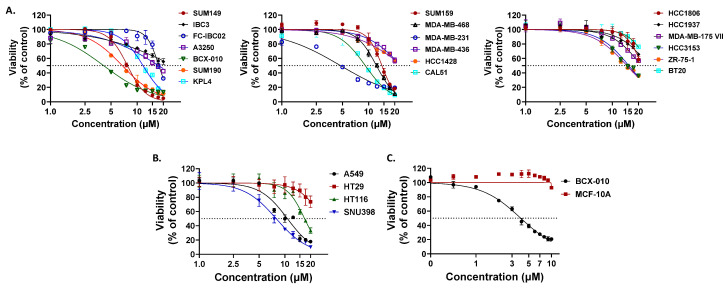
Compound **6** has an antiproliferative effect in cancer cell lines. Sulforhodamine B assay was conducted for 5 days of incubation. (**A**,**C**) Breast cancer cell lines, (**B**) lung (A549), colon (HT29, HT116), and liver (SNU398) cancer cell lines.

Flavonoids, a diverse group of plant-derived polyphenolic compounds, are well documented for their broad-spectrum biological activities, including antioxidant, anti-inflammatory, antimicrobial, and anticancer properties [41,42,43]. To evaluate the antitumor potential of thirteen compounds extracted from *D. viscosa* flowers, we performed in vitro proliferation assays using the SUM149 triple-negative breast cancer (TNBC) cell line. Among the tested compounds, compound **6** (santin) exhibited the dose-dependent inhibition of cell growth (Figure 2), while the remaining twelve compounds showed no significant inhibitory effect at concentrations up to 20 μM. We further assessed the therapeutic potential of compound **6** across various breast cancer subtypes and other cancer cell lines. Compound **6** demonstrated dose-dependent growth inhibition in breast cancer cell lines, including TNBC (IC_50_: 4.22–26.71 µM), HER2-positive (HER2+) (IC_50_: 6.74–25.61 µM), and hormone receptor-positive (HR+) (IC_50_: 14.71–24.35 µM) cell lines (Figure 3A). Notably, inflammatory breast cancer (IBC) cell lines, which represent the most aggressive and lethal subtypes, showed heightened sensitivity to compound **6**. The lowest IC₅₀ values were observed in BCX-010 (4.22 µM), SUM190 (6.74 µM), and SUM149 (7.73 µM), compared to non-IBC cell lines. In addition to breast cancer models, compound 6 also showed antitumor activity in non-small cell lung cancer (A549, IC_50_ = 10.73 µM), colon cancer (HT29, IC_50_ = 27.88 µM; HT116, IC_50_ = 17.03 µM), and hepatocellular carcinoma (SNU398, IC_50_ = 7.39 µM) (Figure 3B). Importantly, compound **6** displayed minimal cytotoxicity in the normal mammary epithelial cell line MCF-10A (IC_50_ > 20 µM), suggesting a favorable therapeutic index and minimal toxicity to normal tissues (Figure 3C). The IC_50_ values for all tested cell lines are summarized in Table 2.

**Figure 4:**
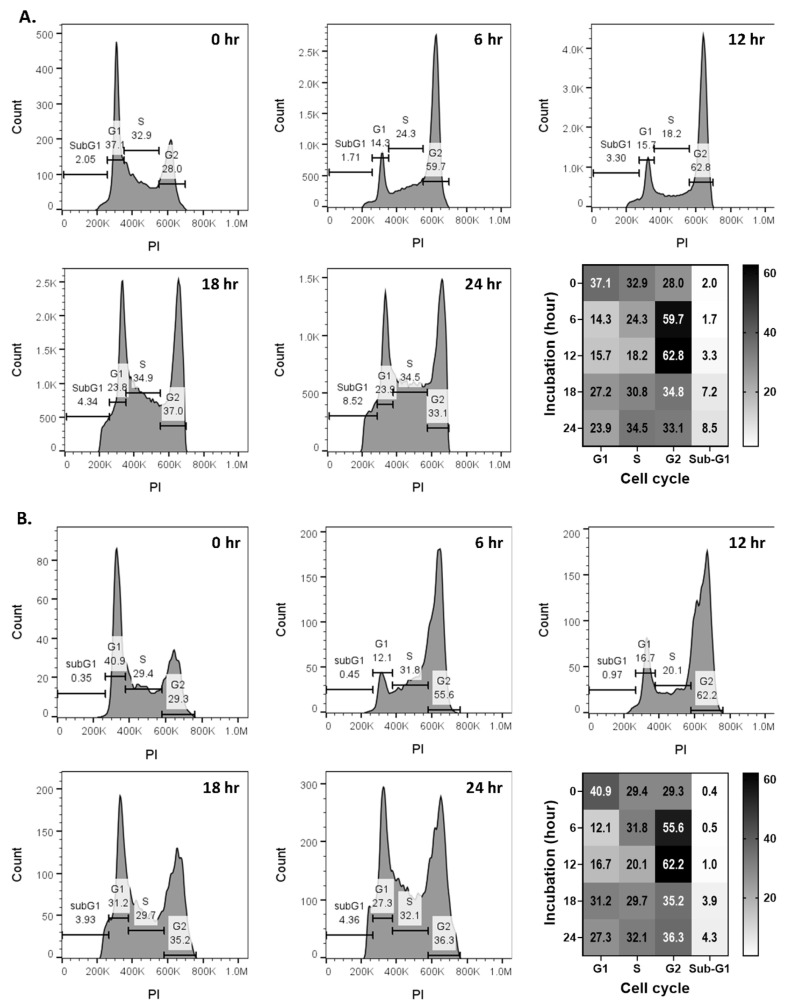
Compound **6** induces G2-M cell cycle arrest. Cells were treated with compound **6** for the indicated time and then analyzed by flow cytometry. Heatmaps were generated using the Graph Pad Prism software package (V10). (**A**) SUM149 and (**B**) BCX-010.

**Figure 5:**
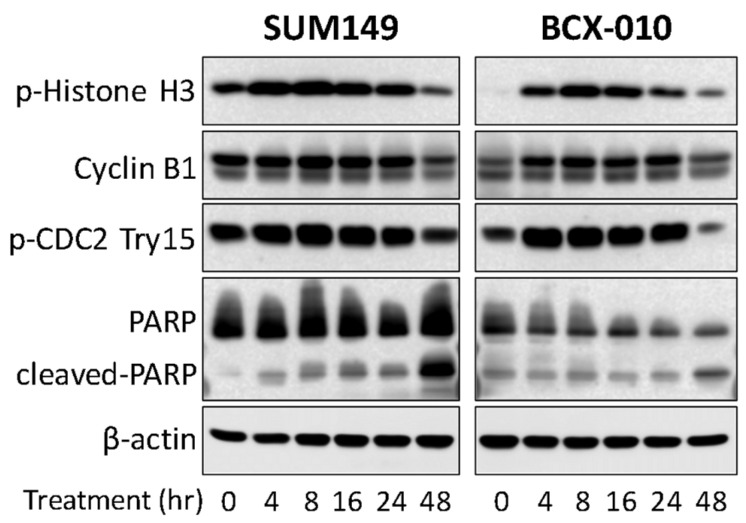
Activation of G2-M Checkpoint and induction of apoptosis by compound **6**. Western blotting: The breast cancer cell lines were treated with IC_50_ concentration of compound **6** for the indicated time points, and then whole cell lysates were collected for Western blotting.

To investigate the mechanism of action, we examined cell cycle progression in SUM149 and BCX-010 IBC cell lines. Compared to control cells, compound **6** treatment led to a marked accumulation of cells in the G2-M phase arrest within 6–12 h, ranging from 59.7% to 62.8% in SUM149 cells (Figure 4A) and 55.6% to 62.2% in BCX-010 cells (Figure 4B). To further validate G2–M checkpoint arrest, we performed a Western blot analysis to assess the expression of key cell cycle regulatory proteins. As shown in Figure 5, compound **6** induced a time-dependent increase in phospho-Histone H3 (a mitotic marker indicating metaphase accumulation) and phospho-CDC2 (Tyr15), a negative regulator of CDK1 activity. These findings confirm that compound **6** causes arrest in both the G2 phase and mitosis. Additionally, we observed an increase in the apoptosis marker cleaved PARP, suggesting that G2–M arrest induced by compound **6** ultimately leads to apoptotic cell death.

Flavonoids, specifically the subclass flavanones, are characterized by the absence of a double bond between the C–2 and C–3 positions. This structural difference results in losing the planar configuration of the rings in the benzo-γ-pyrone molecules, which is associated with a decrease in cytotoxicity [44]. Supporting this structure–activity relationship (SAR), flavanones **9**–**12**, isolated from the flower extract of *D. viscosa*, showed no activity against all tested inflammatory (IBC) breast cancer cell lines.

Quercetin and kaempferol are well-studied flavonols that exhibit anticancer and antioxidant properties in several cancer cell lines. Studies in the literature have shown that flavanols differ from flavones, in which the presence of a hydroxy group at the C–3 position is linked to reduced cytotoxicity. For example, kaempferol and quercetin exhibited less pronounced cytotoxicity compared to apigenin [45]. Supporting this SAR, flavanols quercetin (**1**), kaempferol (**2**), kaempferide (**3**), 3,5-dihydroxy-7,4′-dimethoxyflavone (**4**), mikanin (**5**), and 6-methoxykaempferol (**7**), which were isolated from the flowers extract of *D. viscosa*, demonstrated little to no activity against the tested IBC breast cancer cell lines, highly aggressive subtypes.

Flavones isolated from *D. vicosa* flowers, namely makinin (**5**), santin (**6**), and 5-hydroxy-3,7,4-trimethoxyflavone (**8**), demonstrate varying levels of activity. Notably, compounds **5** and **8** show lower or no activity when compared to compound **6**, which possesses a hydroxyl group at position C7. The presence of a hydroxyl group at C7 is essential for the cytotoxic activity observed in cancer cell lines [33]. The incorporation of methoxy groups in flavones decisively enhances their lipophilicity and membrane permeability, facilitating greater accumulation within cells. Our structure–activity relationship (SAR) analysis clearly reveals that 3,6-dimethoxyflavanone from santin (**6**) exhibits significant antiproliferative activity against IBC cell lines. While methoxy groups may contribute to metabolic challenges, they also confer selectivity and potent anti-inflammatory effects in flavone analogs. Specifically, santin effectively promotes TRAIL-mediated apoptosis in colon cancer cells, highlighting its potential as a therapeutic agent [34].

## 3. Materials and Methods

### 3.1. General Experimental Procedures

NMR spectra were recorded on a Bruker AVANCE DRX400 NMR spectrometer (Bruker, Billerica, MA, USA) at 400 MHz for ^1^H and 100 MHz for ^13^C. Data processing was performed using MestReNova 14.2.1-27684, with CDCl_3_ (*δ*_H_ 7.23, *δ*_C_ 77.16) or MeOD (*δ*_H_ 3.31, *δ*_C_ 49.0) as solvents. Silica gel (230–400 mesh, 480–800 mesh, Sorbent Technologies, Atlanta, GA, USA), C-18 reversed-phase (particle size 40–63 μm, Sorbent technologies, USA), and Sephadex LH-20 (GE Healthcare, Piscataway, NJ, USA) were used for column chromatography (CC). ACS grade solvent (acetone, hexane, ethyl acetate, methanol) used for extraction, isolation was analytical grade (VWR, Radnor, PA, USA). An amount of 10% cerium (IV) sulfate solution (Cat. No. 13590-82-4, Aldrich, St. Louis, MO, USA) in 15% sulfuric acid (Cat. No. 339741, Aldrich, St. Louis, MO, USA) was used as a TLC reagent.

### 3.2. Plant Materials

On 31 May 2023, fresh flowers of *D. viscosa* were collected from Hawaii Volcanoes National Park, located at 19.45482′ N, 155.33612′ W, along Mauna Loa Road, Hawai‘i, USA. The collected flowers were cleaned to remove dust and dirt before extraction. The plant was identified by Kumu Piilani Kaawaloa, and a voucher specimen (No. MQF05) was deposited at the Natural Product Chemistry Laboratory in the Department of Pharmaceutical Sciences at Daniel K. Inouye College of Pharmacy, University of Hawai‘i at Hilo.

### 3.3. Extraction and Isolation

The fresh flowers of *D. viscosa* (203.4 g) were finely ground and extracted with three times two liters of methanol for three days at room temperature. The extract was filtered through filter paper (qualitative 2, Cat. No. 1002110, Whatman, Buckinghamshire, UK). After solvent removal using a rotary evaporator, a methanol crude extract weighing 25.39 g was obtained. The methanol extract (25.39 g) was subjected to reversed-phase C-18 column chromatography (CC) [particle size 40–63 μm, Cat. No. 52546M-05, Sorbent technologies, USA] using a gradient elution of H_2_O-MeOH-acetone (1:0:0 to 0:0:1, *v*/*v*), yielding 21 fractions (A-W). Compounds **3** (184.4 mg), **10** (148.6 mg), and **13** (8.7 mg) were isolated directly. Fractions D, E, F, and G were combined (7.8 g) and further separated by Sephadex-LH20 CC (100% MeOH), yielding compounds **1** (6.4 mg), **2** (6.1 mg), **7** (282.3 mg), **9** (15.3 mg), and **11** (108.4 mg). Fraction K (264.8 mg) was purified by Sephadex-LH20 CC (100% MeOH) followed by silica gel CC (20% acetone-hexanes), yielding compounds **5** (1.6 mg), **6** (8.4 mg), and **14** (3.4 mg). Fraction N (1.8 g) was purified by Sephadex-LH20 CC (100% MeOH) followed by silica gel CC (20% ethyl acetate-hexanes), yielding compounds **3** (3.1 mg), **4** (12.4 mg), **8** (3.5 mg), and **12** (132.3 mg).

*Quercetin* (**1**): yellow solids, mp 315–316 °C; ^1^H NMR (acetone-*d*_6_, 400 MHz) *δ* 12.18 (1H, s, 5-OH), 7.80 (1H, d, *J* = 2.0 Hz, H-2′), 7.68 (1H, dd, *J* = 8.4, 2.0 Hz, H-6′), 6.97 (1H, d, *J* = 8.4 Hz, H-5′), 6.52 (1H, d, *J* = 2.0 Hz, H-8), and 6.25 (1H, d, *J* = 2.0 Hz, H-6).
*Kaempferol* (**2**): yellow solids, mp 277–278 °C; ^1^H NMR (MeOD, 400 MHz) *δ* 8.08 (2H, d, *J* = 8.9 Hz, H-2′ and H-6′), 6.91 (2H, d, *J* = 8.9 Hz, H-3′ and H-5′), 6.38 (1H, d, *J* = 2.0 Hz, H-8), and 6.17 (1H, d, *J* = 2.0 Hz, H-6).
*Kaempferide* (**3**): yellow solids, mp 228–229 °C; ^1^H NMR (acetone-*d*_6_, 400 MHz) *δ* 12.16 (1H, br s, 5-OH), 8.21 (2H, d, *J* = 8.8 Hz, H-2′ and H-6′), 7.10 (2H, d, *J* = 8.8 Hz, H-3′ and H-5′), 6.53 (1H, d, *J* = 2.0 Hz, H-8), 6.26 (1H, d, *J* = 2.0 Hz, H-6), and 3.89 (3H, s, CH_3_-4′).
*3,5-Dihydroxy-7,4′-dimethoxyflavone* (**4**): yellow solids, mp 158–159 °C; ^1^H NMR (CDCl_3_, 400 MHz) *δ* 11.74 (1H, br s, 5-OH), 8.28 (2H, d, *J* = 8.8 Hz, H-2′ and H-6′), 7.04 (2H, d, *J* = 8.8 Hz, H-3′ and H-5′), 6.48 (1H, d, *J* = 2.0 Hz, H-8), 6.37 (1H, d, *J* = 2.0 Hz, H-6), and 3.89 (6H, s, CH_3_-7 and CH_3_-4′).
*Mikanin* (**5**): yellow solids, ^1^H NMR (CDCl_3_, 400 MHz) *δ* 11.83 (1H, br s, 5-OH), 8.19 (2H, d, *J* = 8.7 Hz, H-2′ and H-6′), 7.03 (2H, d, *J* = 8.7 Hz, H-3′ and H-5′), 6.55 (1H, s, H-8), 3.97 (3H, s, CH_3_-7), 3.93 (3H, s, CH_3_-6), and 3.89 (3H, s, CH_3_-4′).
*Santin* (**6**): yellow solids, mp 157–158 °C; ^1^H NMR (CDCl_3_, 400 MHz) *δ* 12.90 (1H, br s, 5-OH), 8.08 (2H, d, *J* = 9.0 Hz, H-2′ and H-6′), 7.04 (2H, d, *J* = 9.0 Hz, H-3′ and H-5′), 6.58 (1H, s, H-8), 4.07 (3H, s, CH_3_-6), 3.92 (3H, s, CH_3_-4′), and 3.88 (3H, s, CH_3_-3).
*6-Methoxykaempferol* (**7**): yellow solids, mp 239–240 °C; ^1^H NMR (MeOD, 400 MHz) *δ* 8.09 (2H, d, *J* = 9.0 Hz, H-2′ and H-6′), 6.91 (2H, d, *J* = 9.0 Hz, H-3′ and H-5′), 6.52 (1H, s, H-8), and 3.90 (3H, s, CH_3_-6).
*5-Hydroxy-3,7,4′-trimethoxyflavone* (**8**): yellow solids, mp 140–141 °C; ^1^H NMR (CDCl_3_, 400 MHz) *δ* 12.67 (1H, br s, 5-OH), 8.11 (2H, d, *J* = 9.0 Hz, H-2′ and H-6′), 7.05 (2H, d, *J* = 9.0 Hz, H-3′ and H-5′), 6.46 (1H, d, *J* = 2.0 Hz, H-8), 6.37 (1H, d, *J* = 2.0 Hz, H-6), 3.92 (3H, s, CH_3_-7), 3.90 (3H, s, CH_3_-4′), and 3.88 (3H, s, CH_3_-3).
*Naringenin* (**9**): pale-yellow solids, mp 250–251 °C; ^1^H NMR (MeOD, 400 MHz) *δ* 7.29 (2H, d, *J* = 8.5 Hz, H-2′ and H-6′), 6.82 (2H, d, *J* = 8.5 Hz, H-3′ and H-5′), 5.89 (1H, d, *J* = 2.0 Hz, H-8), 5.88 (1H, d, *J* = 2.0 Hz, H-6), 5.30 (1H, dd, *J* = 13.0, 3.0 Hz, H-2), 3.08 (1H, dd, *J* = 16.8, 13.0 Hz, H-3b), 2.65 (1H, dd, *J* = 16.8, 3.0 Hz, H-3a).
*5-Hydroxy-7,4′-dimethoxyflavone* (**10**): pale-yellow solids, mp 164–165 °C; ^1^H NMR (CDCl_3_, 400 MHz) *δ* 12.06 (1H, s, 5-OH), 7.42 (2H, d, *J* = 8.5 Hz, H-2′ and H-6′), 6.97 (2H, d, *J* = 8.5 Hz, H-3′ and H-5′), 6.09 (1H, d, *J* = 2.0 Hz, H-8), 6.07 (1H, d, *J* = 2.0 Hz, H-6), 5.40 (1H, dd, *J* = 13.0, 3.0 Hz, H-2), 3.86 (3H, s, CH_3_-7), 3.83 (3H, s, CH_3_-4′), 3.09 (1H, dd, *J* = 16.8, 13.0 Hz, H-3b), and 2.79 (1H, dd, *J* = 16.8, 3.0 Hz, H-3a).
*2,3-Dihydro-5,7-dihydroxy-6-methoxy-2-(4-methoxyphenyl)-4H-1-benzopyran-4-one* (**11**): pale-yellow solids, mp 98–99 °C; ^1^H NMR (MeOD, 400 MHz) *δ* 7.26 (2H, d, *J* = 8.5 Hz, H-2′ and H-6′), 6.79 (2H, d, *J* = 8.5 Hz, H-3′ and H-5′), 6.10 (1H, d, *J* = 2.0 Hz, H-8), 5.27 (1H, dd, *J* = 13.0, 3.0 Hz, H-2), 3.83 (3H, s, CH_3_-6), 3.72 (3H, s, CH_3_-4′), 3.05 (1H, dd, *J* = 16.8, 13.0 Hz, H-3b), and 2.64 (1H, dd, *J* = 16.8, 3.0 Hz, H-3a).
*Macarangaflavanone B* (**12**): pale-yellow solids, mp 159–160 °C; ^1^H NMR (MeOD, 400 MHz) *δ* 7.14 (1H, br s, H-2′), 7.09 (1H, br d, *J* = 8.0 Hz, H-6′), 6.77 (1H, d, *J* = 8.0 Hz, H-5′), 5.93 (1H, s, H-8), 5.16 (1H, dd, *J* = 13.0, 3.0 Hz, H-2), 3.02 (1H, dd, *J* = 16.8, 13.0 Hz, H-3b), 2.60 (1H, dd, *J* = 16.8, 3.0 Hz, H-3a), isoprenyl unit at C-6 [*δ*_H_ 5.19 (1H, t, *J* = 7.2 Hz), 3.21 (2H, d, *J* = 7.2 Hz), 1.75 (3H, s), and 1.70 (3H, s)], and isoprenyl unit at C-3′ [*δ*_H_ 5.33 (1H, t, *J* = 7.2 Hz), 3.31 (2H, d, *J* = 7.2 Hz), 1.72 (3H, s), and 1.63 (3H, s)].
*3,5-Diprenyl-4-hydroxybenzaldehyde* (**13**): white solid, mp 84–85 °C; ^1^H NMR (CDCl_3_, 400 MHz) *δ* 9.82 (1H, s, H-7), 7.56 (2H, s, H-2 and H-6), 6.07 (1H, s, 4-OH), 2× isoprenyl unit [*δ*_H_ 5.34 (2H, t, *J* = 7.2 Hz), 3.43 (4H, d, *J* = 7.2 Hz), 1.82 (6H, s), and 1.80 (6H, s)].

### 3.4. Minimum Inhibition Concentration (MIC)

Bacteria strains: The antibacterial activity was tested using selected pathogens and commensal strains from the American Type Culture Collection (ATCC) (Manassas, MD, USA): MRSA USA-300 (LAC, ATCC BAA1756), MRSA N315 (NR-45898), MSSA Newman (ATCC 25904), MSSA 8325-4, *E. coli* (ATCC 9637), *K. pneumoniae* NCTC 9633 (ATCC 13883), and *P. aeruginosa* Boston 41,501 (ATCC 27853). MSSA LUU7, MSSA ONE6, *S. aureus* 8384, MSSA RI27, and MSSA LUE1 were isolated from Hawai’i [46]. *S. aureus* N315 was an MRSA pathogen strain causing hospital-acquired infections in 1982. This strain is easy to acquire antibiotic resistance, such as vancomycin. MSSA strains isolated from the Island of Hawaii include the following: Wailuku River (LUU7); Wailuku River—Estuary (LUE1); Richardson’s Beach Park—Back (RI27); Onekahakaha Beach Park (ONE6) [46]. Mueller–Hinton Broth (MHB, SKU:275730, GTIN:00382902757306, Becton, Dickinson and Company, Franklin Lakes, NJ, USA) and Mueller–Hinton Agar (MHA, SKU:225250, GTIN:00382902252504, Becton, Dickinson and Company, Franklin Lakes, NJ, USA) were used to prepare bacterial inocula. All the inoculate were prepared with fresh cultures plated the day before the test.

The MIC of the samples was determined using a standard two-fold serial dilution method in Mueller–Hinton Broth (MHB, Cat. No. 70192, Sigma-Aldrich, USA), according to guidelines from the Clinical and Laboratory Standards Institute (CLSI) [47,48]. In brief, serial dilutions of the compounds in DMSO were prepared and added to 96-well microplates containing MHB. Different concentrations of the compound (ranging from 0.25 to 128 μg/mL) were then added to each well. Following this, bacterial suspensions (1 × 10^4^ CFU/well) were then inoculated to each well. As a negative control, wells containing 50 μL of MHB broth and 50 μL of bacterial strains without added samples. The plates were incubated at 37 °C for 18–24 h. After incubation, 10 µL of 0.018% resazurin (Cat. No. R7017, Sigma Aldrich, USA) was added to each well. The MIC values were determined after 2–3 h of incubation with resazurin. All antimicrobial assays were performed in triplicate, with vancomycin (Cat. No. 1709007, Sigma Aldrich, USA) and gentamicin (Cat. No. 46305, Sigma Aldrich, USA) used as standard controls.

### 3.5. Cell Lines and Reagents

BT20, HCC1428, HCC3153, HCC1937, HCC1806, MDA-MB-175 VII, MDA-MB-231, MDA-MB-436, MDA-MB-468, A549, HT29, HT116, SNU398, MCF-10A, and ZR-75-1 cells were purchased from American Type Culture Collection (Manassas, VA, USA). SUM149, SUM159, and SUM190 cells were purchased from Asterand Bioscience (now BioIVT, Hicksville, NY, USA). CAL51 cell line was purchased from DSMZ (Braunschweig, Germany). KPL4 cell line was provided by Kawasaki Medical School (Okayama, Japan). A3250 cell line was provided by Icahn School of Medicine at Mount Sinai (New York, NY, USA). FC-IBC02, IBC3, and BCX-010 cell lines were provided by MD Anderson Cancer Center (Houston, TX, USA). HCC1428, HCC3153, HCC1937, HCC1806, MDA-MB-175 VII, A549, HT29, HT116, SNU398, and ZR-75-1 cells were maintained in Roswell Park Memorial Institute 1640 medium (Sigma-Aldrich, St. Louis, MO, USA). BT-20, MDA-MB-231, MDA-MB-436, and MDA-MB-468 cells were maintained in Dulbecco’s modified Eagle’s medium/F-12 medium (DMEM/F-12, Sigma-Aldrich). FC-IBC02, IBC3, BCX-010, SUM190, and KPL4 cells were maintained in Ham’s F-12 medium (Sigma-Aldrich) supplemented with 5 µg/mL of insulin (Thermo Fisher Scientific Inc., Waltham, MA, USA), and 1 µg/mL of hydrocortisone (Sigma-Aldrich). MCF-10A was maintained in DMEM/F-12 medium supplemented with 5% horse serum, 10 µg/mL human insulin, 20 ng/mL EGF, 100 ng/mL Cholera toxin, and 0.5 µg/mL Hydrocortisone (Sigma-Aldrich). All media were supplemented with 10% fetal bovine serum (GenDEPOT, Katy, TX, USA) and 1% antibiotic/antimycotic (Sigma-Aldrich). All cell lines were validated by DNA typing at the MD Anderson Characterized Cell Line Core and confirmed to be free of *Mycoplasma* using the MycoAlert Mycoplasma Detection Kit (Lonza, Morristown, NJ, USA).

### 3.6. Sulforhodamine B Cell Proliferation Assay: Anticancer Activity

The antiproliferative effects of the extract of and compounds isolated from *D. viscosa* on breast cancer cells were evaluated using sulforhodamine B staining assays [49]. Briefly, cells (3 to 10 × 10^3^ cells/well) were seeded in 96-well plates and allowed to adhere overnight. On the following day, cells were treated with samples for 5 days. After the treatment period, cells were fixed with 5% trichloroacetic acid (Cat. No. T6399, Sigma-Aldrich) at 4 °C for 2 h and washed with water three times. Fixed cells were stained with 0.03% sulforhodamine B (Cat. No. 230162, Sigma-Aldrich) solution in 1% acetic acid (Cat. No. 64-19-7, Sigma-Aldrich) for 30 min at room temperature. Excess stain was removed by washing the wells three times with 1% acetic acid. The stained cells were dissolved in 10 mM Tris buffer (Cat. No. T8101-010, GenDEPOT), and the optical density was measured at the wavelength of 560 nm using a Spark enhanced microplate reader (TECAN, Männedorf, Switzerland). The cell proliferation rate was calculated and summarized using descriptive statistics (mean) with the GraphPad Prism software program (version 10; GraphPad Software, Boston, MA, USA).

### 3.7. Cell Cycle Analyses

Breast cancer cells (5 × 10^5^ cells) were placed in a 6 cm culture dish and cultured overnight. The cells were treated with the compound **6** with an IC_50_ concentration for the indicated time points. After incubation, cells were harvested and fixed for DNA content analysis. Adherent cells were detached using trypsin-EDTA and collected into 5 mL conical tubes, followed by centrifugation at 1500 rpm for 4 min at 4 °C. The cell pellet was washed once with 3 mL of ice-cold PBS and centrifuged under the same conditions. The cell pellet was gently resuspended in 350 μL of cold PBS, and then ice-cold 99.9% ethanol (1 mL, 200 proof) was added dropwise to the cell suspension while gently vortexing to avoid cell clumping. Fixed cells were incubated at 4 °C for at least 2 h. The ethanol-fixed cells were centrifuged at 1500 rpm for 4 min and washed twice with 3 mL of cold PBS. The cell pellet was then resuspended in 200 μL of PBS containing 100 μg/mL RNase A and 50 μg/mL propidium iodide (#P4864; Sigma-Aldrich) and incubated at 37 °C for 30 min in the dark. After incubation, samples were analyzed using a Thermo Fisher Attune NxT BRYV6 device (Thermo Fisher Scientific Inc.), and then the cell cycle was determined using FlowJo software v10 (BD Biosciences, Milpitas, CA, USA).

### 3.8. Western Blotting

The effect of compound **6** on the expression of G2–M phase cell cycle-related proteins in breast cancer cell lines was analyzed by Western blotting. Cells were seeded in 6 cm culture dishes and incubated overnight. The following day, cells were treated with compound **6** at its IC_50_ concentration for the indicated time points. Whole-cell lysates were prepared using M-PER lysis buffer (Fisher Scientific) supplemented with protease and phosphatase inhibitors (GenDepot). Protein concentrations were determined, and 20 µg of denatured protein per sample was resolved by SDS-PAGE and transferred onto PVDF membranes (Bio-Rad, Hercules, CA, USA). Membranes were probed with the following primary antibodies at 1:1000 dilution: cleaved PARP (9542S, Cell Signaling Technology, Danvers, MA, USA), phospho-Histone H3 (Ser10; 382159, Cell Signaling Technology), Cyclin B1 (122231, Cell Signaling Technology), phospho-CDC2 (Tyr15; 9114S, Cell Signaling Technology), and β-actin (A5316, Sigma-Aldrich) as a loading control. After washing, membranes were incubated with HRP-conjugated secondary antibodies (1:10,000; Life Technologies, Waltham, MA, USA) and developed using enhanced chemiluminescence. Chemiluminescent signals were detected using the Azure 500 Imaging System (Azure Biosystems, Dublin, CA, USA).

### 3.9. Statistical Analysis

Cell proliferation data were summarized using descriptive statistics (mean), and the IC_50_ values were determined by fitting a dose–response curve using nonlinear regression analysis in GraphPad Prism (version 10, GraphPad Software, Boston, MA, USA).

## 4. Conclusions

*D. viscosa* flowers yielded bioactive compounds with antibacterial and anticancer properties. Macarangaflavanone B (**12**) showed potent antibacterial effects, while santin (**6**) demonstrated selective antiproliferative activity against IBC cell lines. These findings highlight the therapeutic potential of *D. viscosa* and underscore the need for further studies to explore its mechanisms and optimize its use in translational applications. Compounds **5** and **8** are inactive, etc. Santin demonstrated notable antiproliferative activity against IBC cell lines, highlighting its potential as a therapeutic candidate for aggressive cancer subtypes. Its structural features, particularly the hydroxyl group at the C7 position, may play a key role in its cytotoxic effects. While flavonoids such as compounds **5** and **8** exhibited minimal or no activity, the structural characteristics of santin enhance its therapeutic potential. These findings support further investigation of santin in preclinical breast cancer models.

## Figures and Tables

**Table 1:** MIC values (µg/mL) of compounds from *D. viscosa* extract.

Compounds	Gram-Positive									Gram-Negative		
	MRSA USA-300	MSSA LUU7	MSSA 8325-4	*S. aureus* 8384	MSSA ONE6	MSSA RI27	MSSA LUE1	MRSA N315	MSSA Newman	*E. coli*	*K.* *pneumoniae*	*P. aeruginosa*
*D. viscosa* flowers extract	640	320	640	640	320	320	320	320	320	1280	1280	1280
**1**	128	128	128	128	128	128	128	128	128	128	128	128
**2**	64	64	64	64	64	64	64	64	64	128	128	128
**3**	-	-	-	-	-	-	-	-	-	-	128	-
**4**	-	-	-	-	-	-	-	-	-	-	128	128
**5**	-	-	-	-	-	-	-	-	-	-	128	-
**6**	-	-	-	-	-	-	-	-	-	-	128	-
**7**	-	-	-	-	-	-	-	-	-	-	128	128
**8**	-	-	-	-	-	-	-	-	-	-	-	128
**9**	-	-	-	-	-	-	-	-	-	-	-	128
**10**	-	-	-	-	-	-	-	-	-	-	128	128
**11**	-	-	-	-	-	-	-	-	-	128	128	128
**12**	2	2	2	2	2	2	2	2	2	128	128	128
**13**	-	-	-	-	-	-	-	-	-	-	128	128
Vancomycin	0.5	1	0.5	0.5	0.5	1	0.25	0.5	0.5	nt	nt	nt
Gentamicin	nt	nt	nt	nt	nt	nt	nt	nt	nt	0.25	1	0.25

“-” = Inactive indicated a MIC value > 128 µg/mL; nt = not tested.

**Table 2:** IC_50_ values of compound **6** from *D. viscosa* extract.

Cell Lines	Subtype	IC_50_ ± S.D (µM)	Cell Lines	Tumor Type	IC_50_ ± S.D (µM)	Cell Lines	Tumor Type	IC_50_ ± S.D (µM)
SUM149	TNBC	7.73 ± 0.24	HCC1806	TNBC	21.96 ± 1.29	SUM159	TNBC	14.23 ± 0.48
IBC3	HER2+	25.61 ± 3.36	HCC1937	TNBC	26.28 ± 3.43	MDA-MB-468	TNBC	12.3 ± 0.30
FC-IBC02	TNBC	17.81 ± 1.96	MDA-MB-175 VII	HR+ BC	21.48 ± 1.48	MDA-MB-231	TNBC	4.96 ± 0.44
A3250	TNBC	17.63 ± 1.84	HCC3153	TNBC	15.46 ± 0.66	A549	NSCLC	10.73 ± 0.81
BCX-010	TNBC	4.22 ± 0.37	ZR-75-1	HR+ BC	14.71 ± 0.58	HT29	Colon	27.88 ± 5.43
SUM190	HER2+	6.74 ± 0.32	CAL51	TNBC	9.16 ± 0.40	HT116	Colon	17.03 ± 0.81
KPL4	HER2+	11.69 ± 0.36	HCC1428	HR+ BC	24.35 ± 1.52	SNU398	HCC	7.39 ± 0.54
BT20	TNBC	26.71 ± 3.56	MDA-MB-436	TNBC	23.48 ± 1.16	MCF-10A	Normal	>20

## Data Availability

The data are included in this article.

## Supplementary Materials

The following supporting information can be downloaded at: https://www.mdpi.com/article/10.3390/molecules30112274/s1. Figure S1: ^1^H NMR spectrum (400 MHz, Acetone-*d*_6_) of compound **1**. Figure S2: ^1^H NMR spectrum (400 MHz, MeOD) of compound **2**. Figure S3: ^1^H NMR spectrum (400 MHz, Acetone-*d*_6_) of compound **3**. Figure S4: ^13^C NMR spectrum (100 MHz, Acetone-*d*_6_) of compound **3**. Figure S5: HSQC spectrum of compound **3**. Figure S6: HMBC spectrum of compound **3**. Figure S7: ^1^H NMR spectrum (400 MHz, CDCl_3_) of compound **4**. Figure S8: ^13^C NMR spectrum (100 MHz, CDCl_3_) of compound **4**. Figure S9: HSQC spectrum of compound **4**. Figure S10: HMBC spectrum of compound **4**. Figure S11: ^1^H NMR spectrum (400 MHz, CDCl_3_) of compound **5**. Figure S12: ^13^C NMR spectrum (100 MHz, CDCl_3_) of compound **5**. Figure S13: HSQC spectrum of compound **5**. Figure S14: HMBC spectrum of compound **5**. Figure S15: ^1^H NMR spectrum (400 MHz, CDCl_3_) of compound **6**. Figure S16: ^13^C NMR spectrum (100 MHz, CDCl_3_) of compound **6**. Figure S17: HSQC spectrum of compound **6**. Figure S18: HMBC spectrum of compound **6**. Figure S19: ^1^H NMR spectrum (400 MHz, MeOD) of compound **7**. Figure S20: ^13^C NMR spectrum (100 MHz, MeOD) of compound **7**. Figure S21: HSQC spectrum of compound **7**. Figure S22: HMBC spectrum of compound **7**. Figure S23: ^1^H NMR spectrum (400 MHz, CDCl_3_) of compound **8**. Figure S24: ^13^C NMR spectrum (100 MHz, CDCl_3_) of compound **8**. Figure S25: HSQC spectrum of compound **8**. Figure S26: HMBC spectrum of compound **8**. Figure S27: ^1^H NMR spectrum (400 MHz, MeOD) of compound **9**. Figure S28: ^13^C NMR spectrum (100 MHz, MeOD) of compound **9**. Figure S29: ^1^H NMR spectrum (400 MHz, CDCl_3_) of compound **10**. Figure S30: ^13^C NMR spectrum (100 MHz, CDCl_3_) of compound **10**. Figure S31: HSQC spectrum of compound **10**. Figure S32: HMBC spectrum of compound **10**. Figure S33: ^1^H NMR spectrum (400 MHz, MeOD) of compound **11**. Figure S34: ^13^C NMR spectrum (100 MHz, MeOD) of compound **11**. Figure S35: HSQC spectrum of compound **11**. Figure S36: HMBC spectrum of compound **11**. Figure S37: ^1^H NMR spectrum (400 MHz, MeOD) of compound **12**. Figure S38: ^13^C NMR spectrum (100 MHz, MeOD) of compound **12**. Figure S39: HSQC spectrum of compound **12**. Figure S40: HMBC spectrum of compound **12**. Figure S41: ^1^H NMR spectrum (400 MHz, CDCl_3_) of compound **13**. Figure S42: ^13^C NMR spectrum (100 MHz, CDCl_3_) of compound **13**.
