# Supplementary material for: Bioactive Compounds from Dodonaea viscosa Flowers: Potent Antibacterial and Antiproliferative Effects in Breast Cancer Cells
Source: Molecules. 2025 May 22;30(11):2274. doi: 10.3390/molecules30112274 (PMC12155741; doi:10.3390/molecules30112274)
Supplement: Supplementary file 1 [file molecules-30-02274-s001.zip › molecules-3429462-supplementary.pdf]

SUPPLEMENTARY MATERIALS

Bioactive Compounds from *Dodonaea viscosa* Flowers: Potent Antibacterial and Antiproliferative Effects in Breast Cancer Cells

Achara Raksat [1], Daniel Yee [1], Young Jin Gi [2], Supakit Wongwiwatthananukit [3], Leng Kar Chang [4], Kumu Piilani Kaawaloa [5], Marisa M. Wall [6], Jangsoon Lee [2,]* and Leng Chee Chang [1,]*

[1] Department of Pharmaceutical Sciences, The Daniel K. Inouye College of Pharmacy, University of Hawai'i at Hilo, Hilo, HI 96720, USA; achara@hawaii.edu (A.R.); yeeds@hawaii.edu (D.Y.)
[2] Preclinical Core, Cancer Biology Program, University of Hawai'i Cancer Center, Honolulu, HI 96813, USA; ygi@cc.hawaii.edu
[3] Department of Pharmacy Practice, The Daniel K. Inouye College of Pharmacy, University of Hawai'i at Hilo, Hilo, HI 96720, USA; supakit@hawaii.edu
[4] Georgia Institute of Science and Research Incorporation, Duluth, GA 30096, USA; lchang@gaisr.org
[5] Hawaiian Language/Performing Arts, Kamehameha Schools, Hawai'i Campus, Kea'au, HI 96749, USA; pikaawal@ksbe.edu
[6] Daniel K. Inouye U.S. Pacific Basin Agricultural Research Center, Hilo, HI 96720, USA; marisa.wall@usda.gov
* Correspondence: jlee@cc.hawaii.edu (J.L.); lengchee@hawaii.edu (L.C.C.); Tel.: +1-808-237-3925 (J.L.); +1-808-932-8124 (L.C.C.)

Table of Contents **Page**

**Figure S1**.	^{1}H NMR spectrum (400 MHz, Acetone-d_6) of compound **1**.	3
**Figure S2**.	^{1}H NMR spectrum (400 MHz, MeOD) of compound **2**.	3
**Figure S3**.	^{1}H NMR spectrum (400 MHz, Acetone-d_6) of compound **3**.	4
**Figure S4**.	^{13}C NMR spectrum (100 MHz, Acetone-d_6) of compound **3**.	4
**Figure S5**.	HSQC spectrum of compound **3**.	5
**Figure S6**.	HMBC spectrum of compound **3**.	5
**Figure S7**.	^{1}H NMR spectrum (400 MHz, $CDCl_3$) of compound **4**.	6
**Figure S8**.	^{13}C NMR spectrum (100 MHz, $CDCl_3$) of compound **4**.	6
**Figure S9**.	HSQC spectrum of compound **4**.	7
**Figure S10**.	HMBC spectrum of compound **4**.	7
**Figure S11**.	^{1}H NMR spectrum (400 MHz, $CDCl_3$) of compound **5**.	8
**Figure S12**.	^{13}C NMR spectrum (100 MHz, $CDCl_3$) of compound **5**.	8
**Figure S13**.	HSQC spectrum of compound **5**.	9
**Figure S14**.	HMBC spectrum of compound **5**.	9
**Figure S15**.	^{1}H NMR spectrum (400 MHz, $CDCl_3$) of compound **6**.	10
**Figure S16**.	^{13}C NMR spectrum (100 MHz, $CDCl_3$) of compound **6**.	10
**Figure S17**.	HSQC spectrum of compound **6**.	11
**Figure S18**.	HMBC spectrum of compound **6**.	11
**Figure S19**.	^{1}H NMR spectrum (400 MHz, MeOD) of compound **7**.	12
**Figure S20**.	^{13}C NMR spectrum (100 MHz, MeOD) of compound **7**.	12
**Figure S21**.	HSQC spectrum of compound **7**.	13
**Figure S22**.	HMBC spectrum of compound **7**.	13
**Figure S23**.	^{1}H NMR spectrum (400 MHz, $CDCl_3$) of compound **8**.	14
**Figure S24**.	^{13}C NMR spectrum (100 MHz, $CDCl_3$) of compound **8**.	14
**Figure S25**.	HSQC spectrum of compound **8**.	15
**Figure S26**.	HMBC spectrum of compound **8**.	15
**Figure S27**.	^{1}H NMR spectrum (400 MHz, MeOD) of compound **9**.	16
**Figure S28**.	^{13}C NMR spectrum (100 MHz, MeOD) of compound **9**.	16
**Figure S29**.	^{1}H NMR spectrum (400 MHz, $CDCl_3$) of compound **10**.	17
**Figure S30**.	^{13}C NMR spectrum (100 MHz, $CDCl_3$) of compound **10**.	17
**Figure S31**.	HSQC spectrum of compound **10**.	18
**Figure S32**.	HMBC spectrum of compound **10**.	18
**Figure S33**.	^{1}H NMR spectrum (400 MHz, MeOD) of compound **11**.	19
**Figure S34**.	^{13}C NMR spectrum (100 MHz, MeOD) of compound **11**.	19
**Figure S35**.	HSQC spectrum of compound **11**.	20
**Figure S36**.	HMBC spectrum of compound **11**.	20
**Figure S37**.	^{1}H NMR spectrum (400 MHz, MeOD) of compound **12**.	21
**Figure S38**.	^{13}C NMR spectrum (100 MHz, MeOD) of compound **12**.	21
**Figure S39**.	HSQC spectrum of compound **12**.	22
**Figure S40**.	HMBC spectrum of compound **12**.	22
**Figure S41**.	^{1}H NMR spectrum (400 MHz, $CDCl_3$) of compound **13**.	23

Figure S42. ^{13}C NMR spectrum (100 MHz, $CDCl_3$) of compound **13**. 23

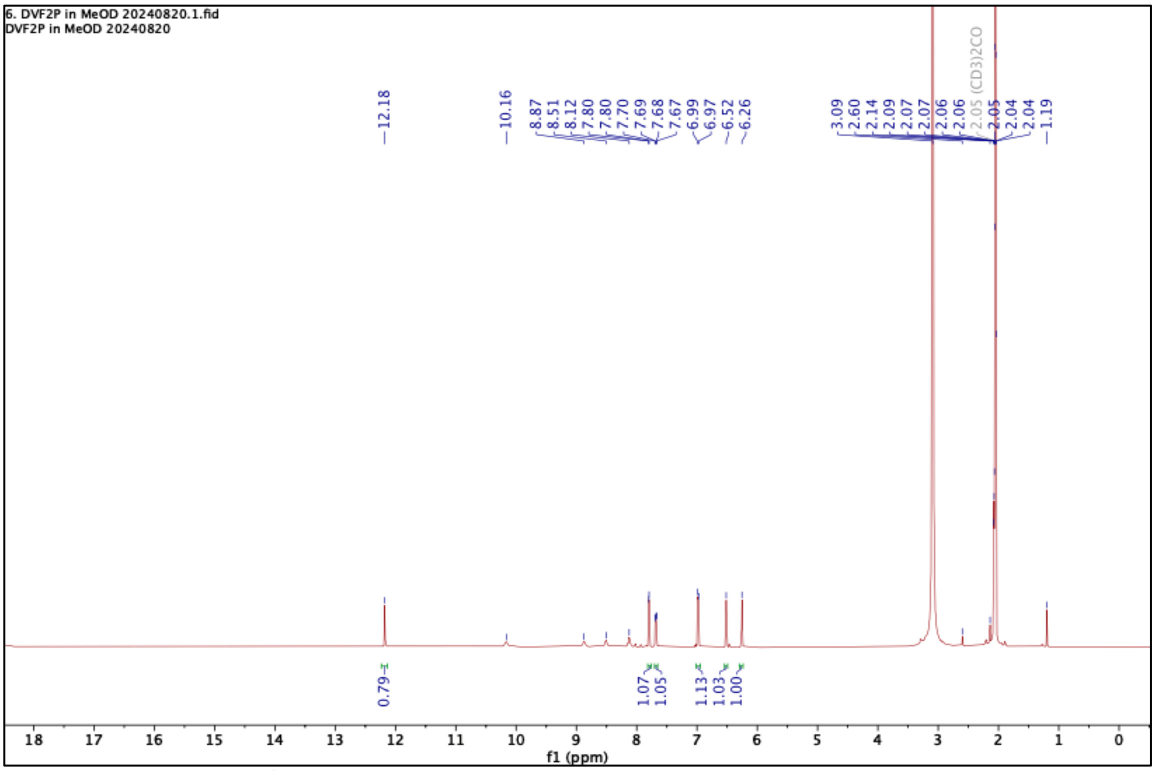

Figure S1. ^{1}H NMR spectrum (400 MHz, Acetone-d_6) of compound **1**.

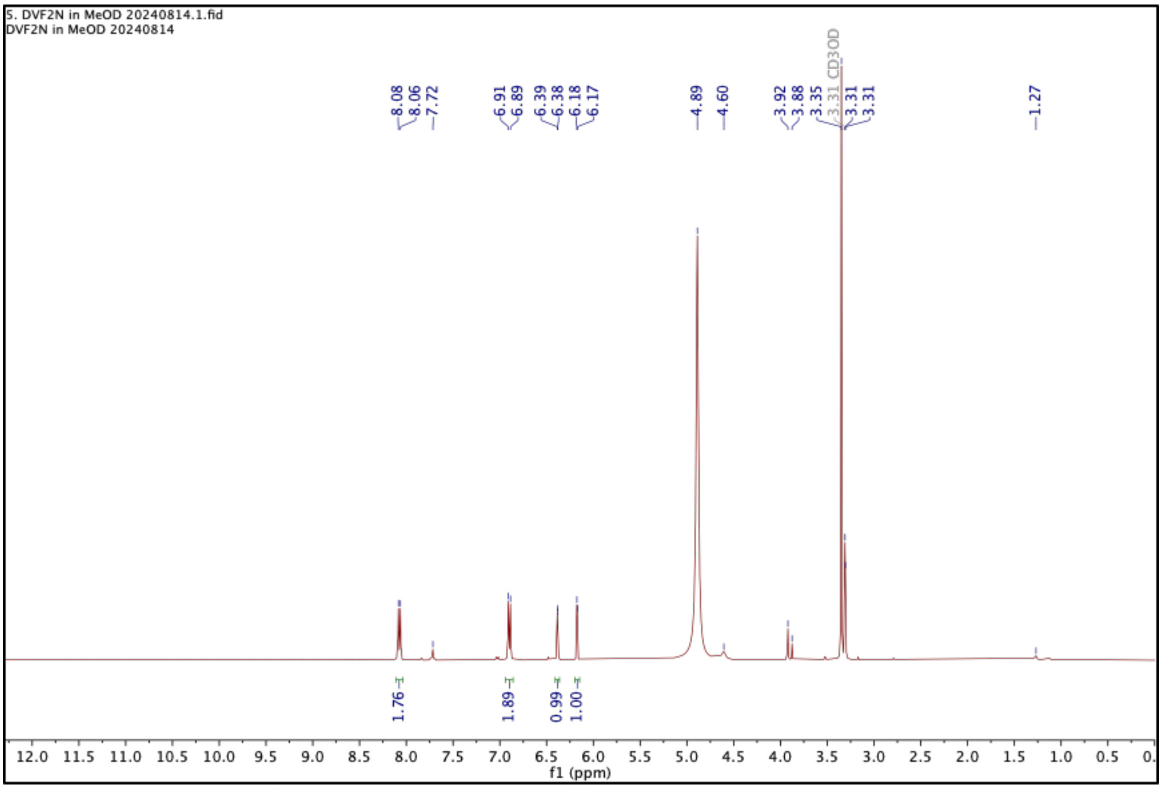

Figure S2. ^{1}H NMR spectrum (400 MHz, MeOD) of compound **2**.

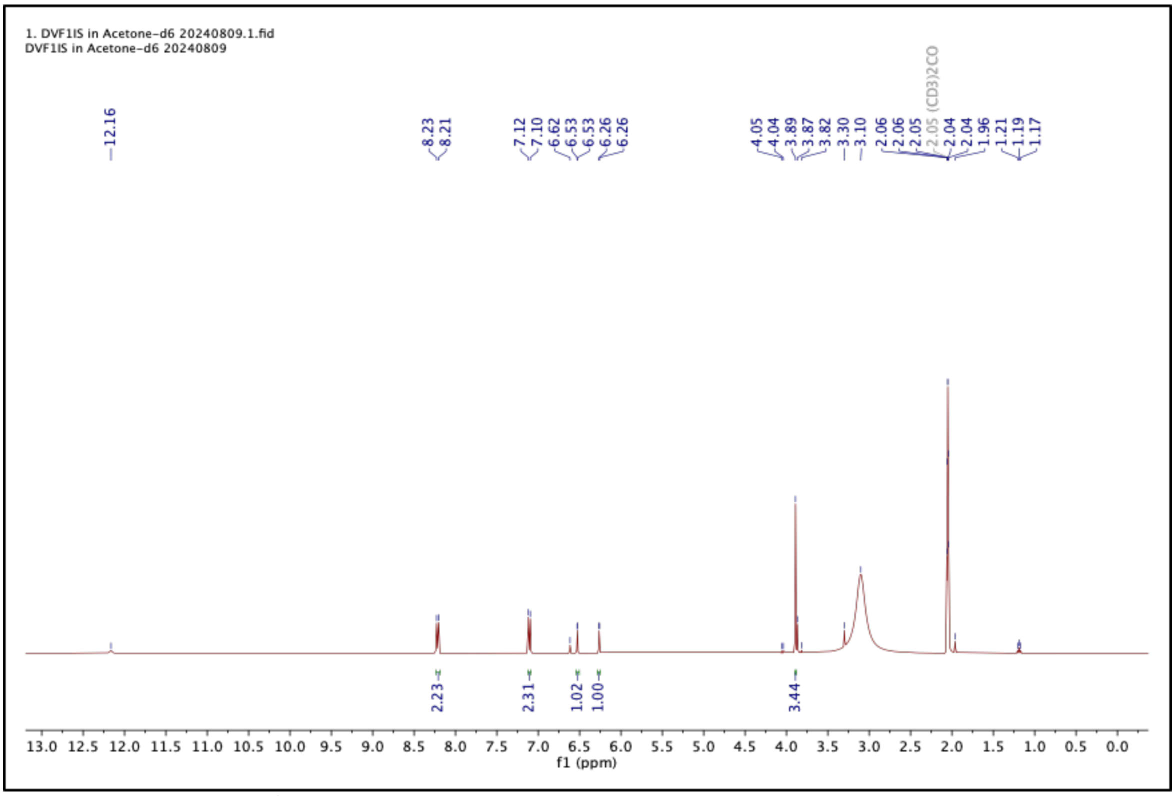

Figure S3. ^{1}H NMR spectrum (400 MHz, Acetone-d$_6$) of compound **3**.

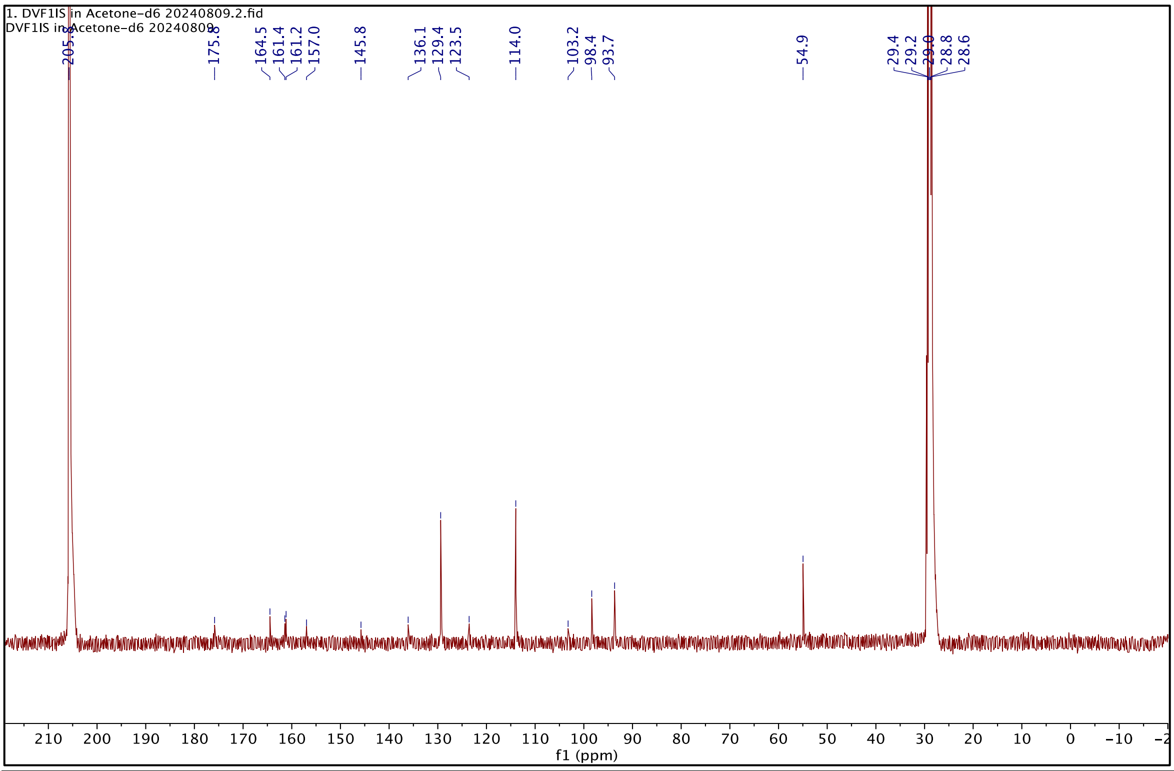

Figure S4. ^{13}C NMR spectrum (100 MHz, Acetone-d$_6$) of compound **3**.

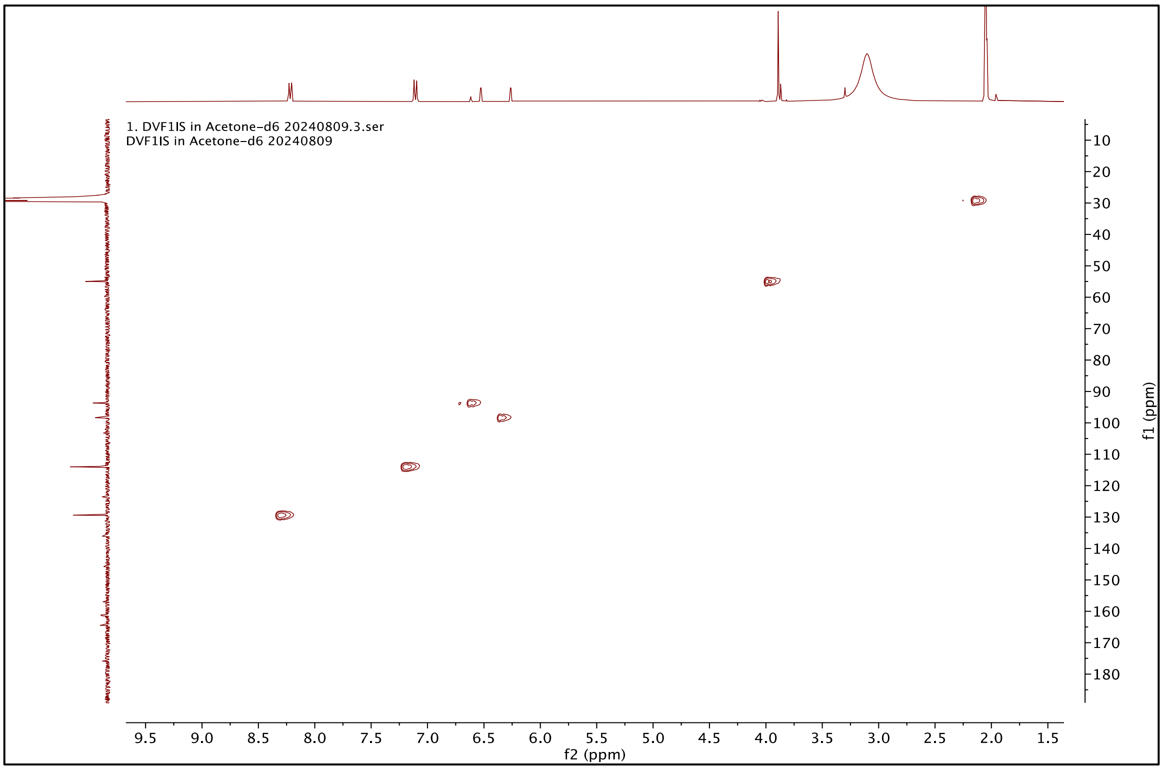

Figure S5. HSQC spectrum of compound **3**.

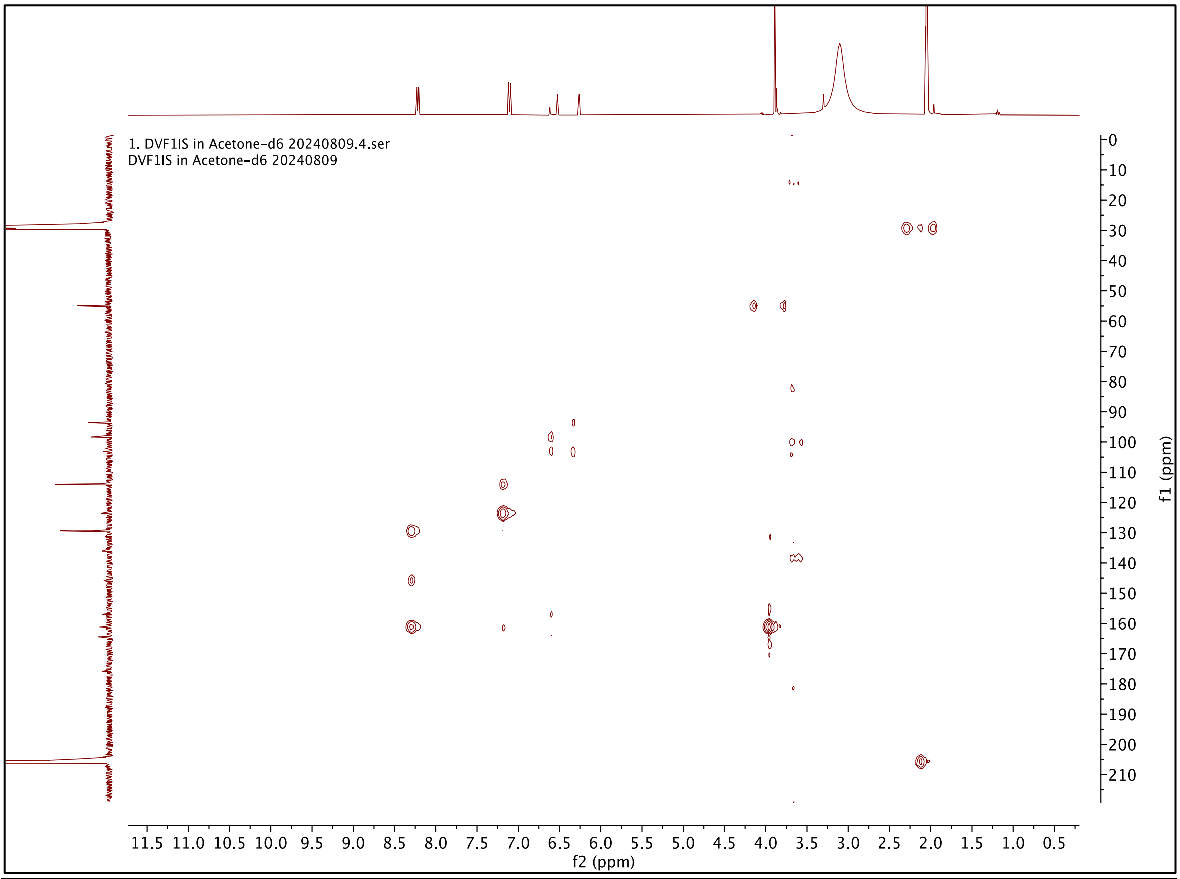

Figure S6. HMBC spectrum of compound **3**.

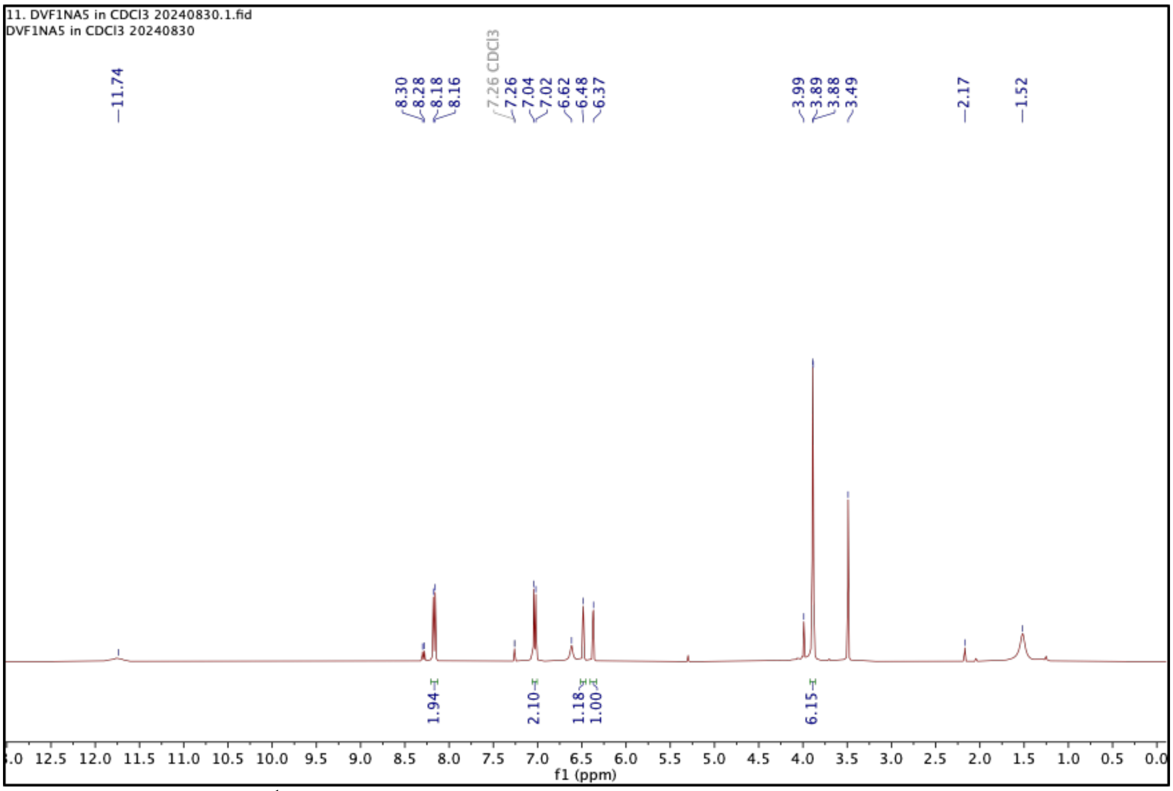

Figure S7. ^{1}H NMR spectrum (400 MHz, $CDCl_3$) of compound **4**.

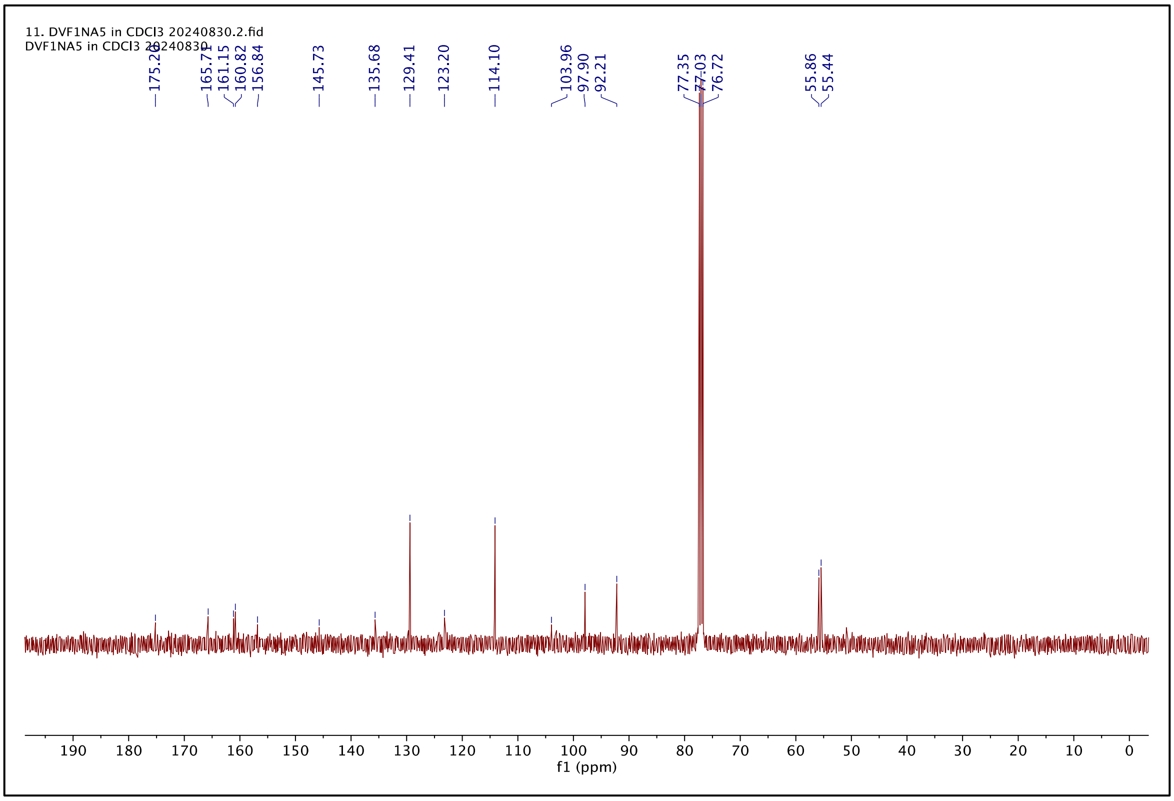

Figure S8. ^{13}C NMR spectrum (100 MHz, $CDCl_3$) of compound **4**.

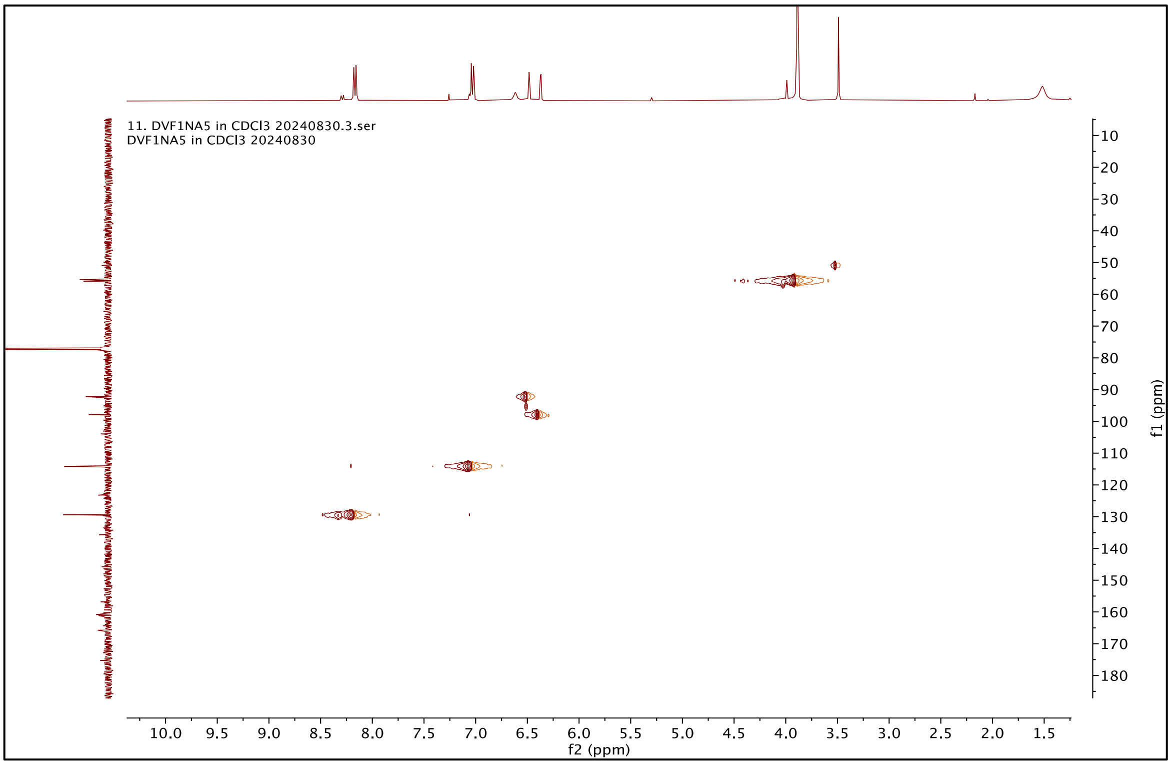

Figure S9. HSQC spectrum of compound **4**.

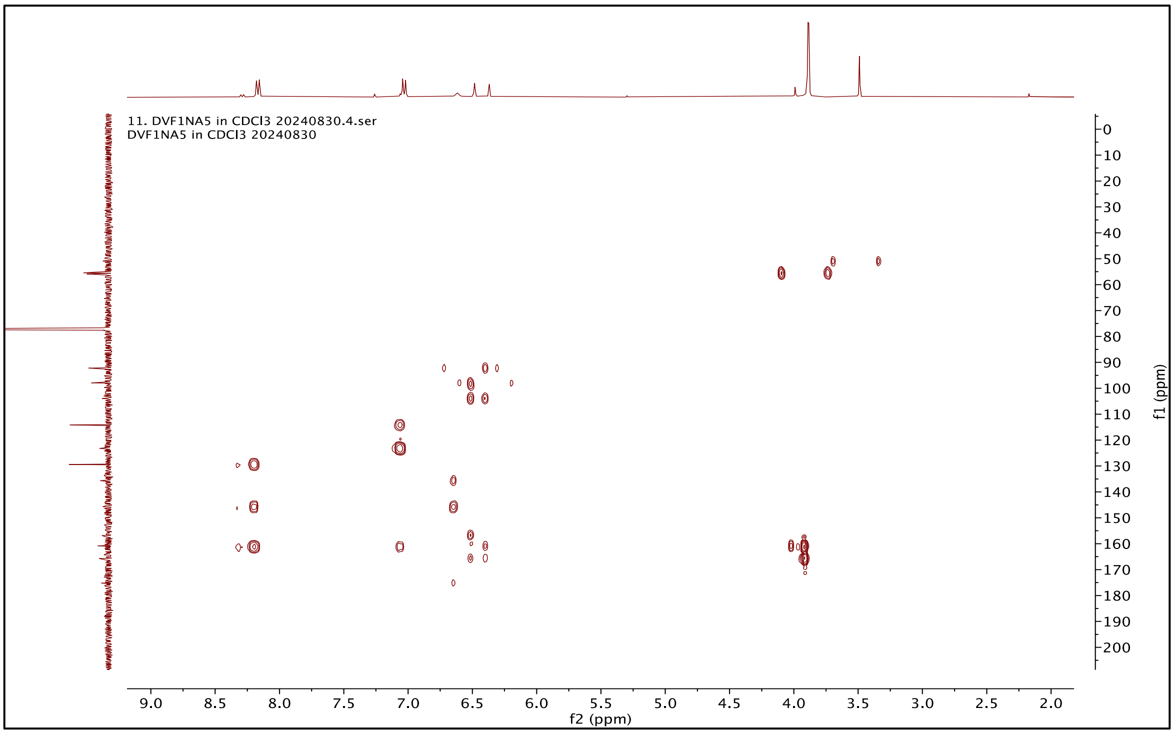

Figure S10. HMBC spectrum of compound **4**.

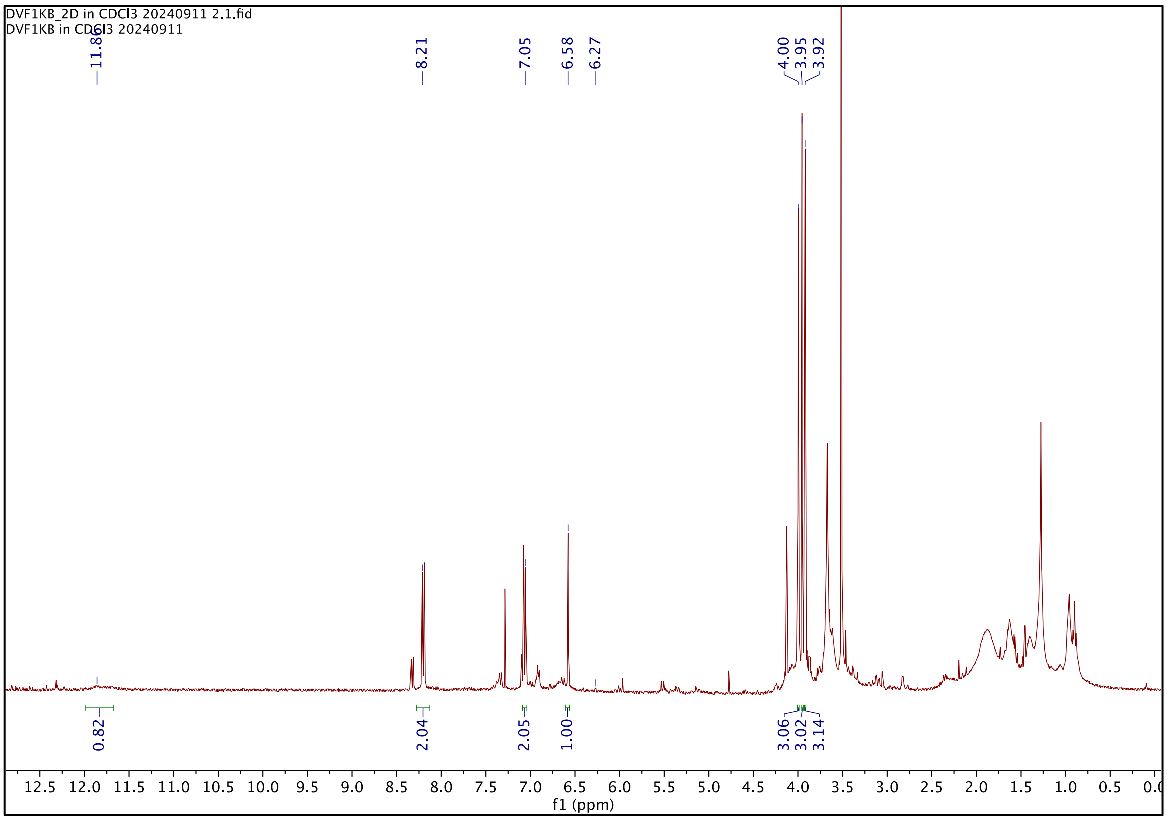

Figure S11. ^{1}H NMR spectrum (400 MHz, $CDCl_3$) of compound **5**.

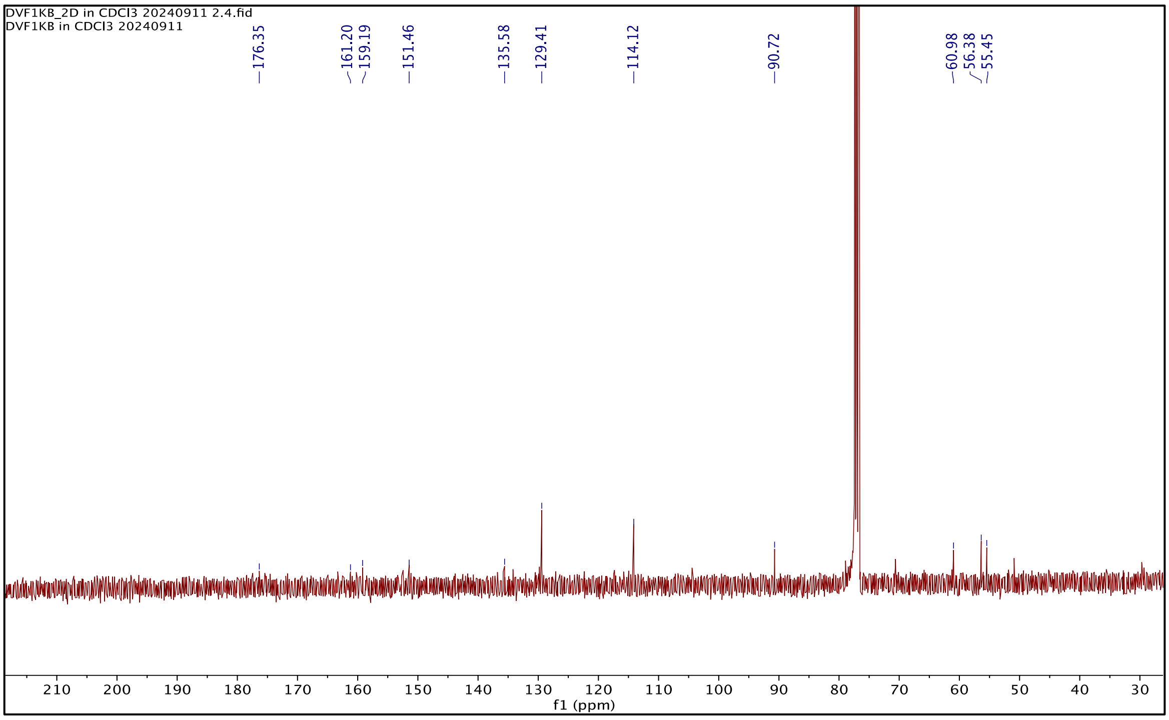

Figure S12. ^{13}C NMR spectrum (100 MHz, $CDCl_3$) of compound **5**.

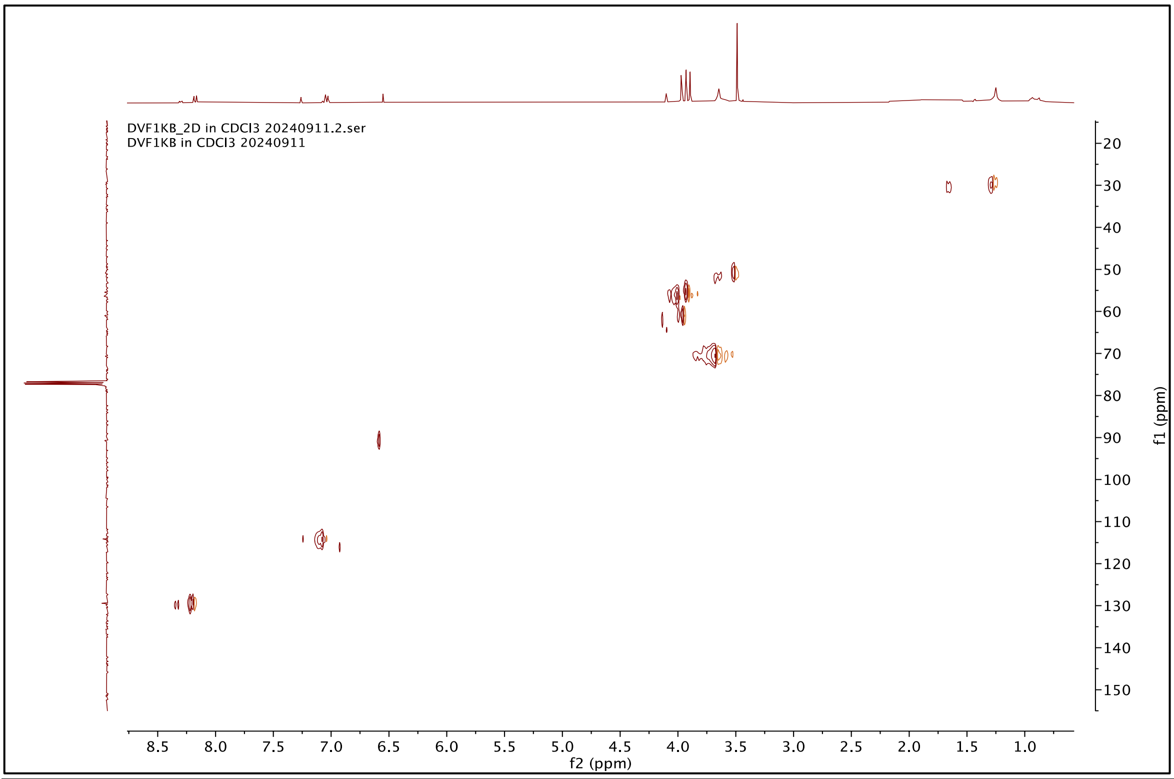

Figure S13. HSQC spectrum of compound **5**.

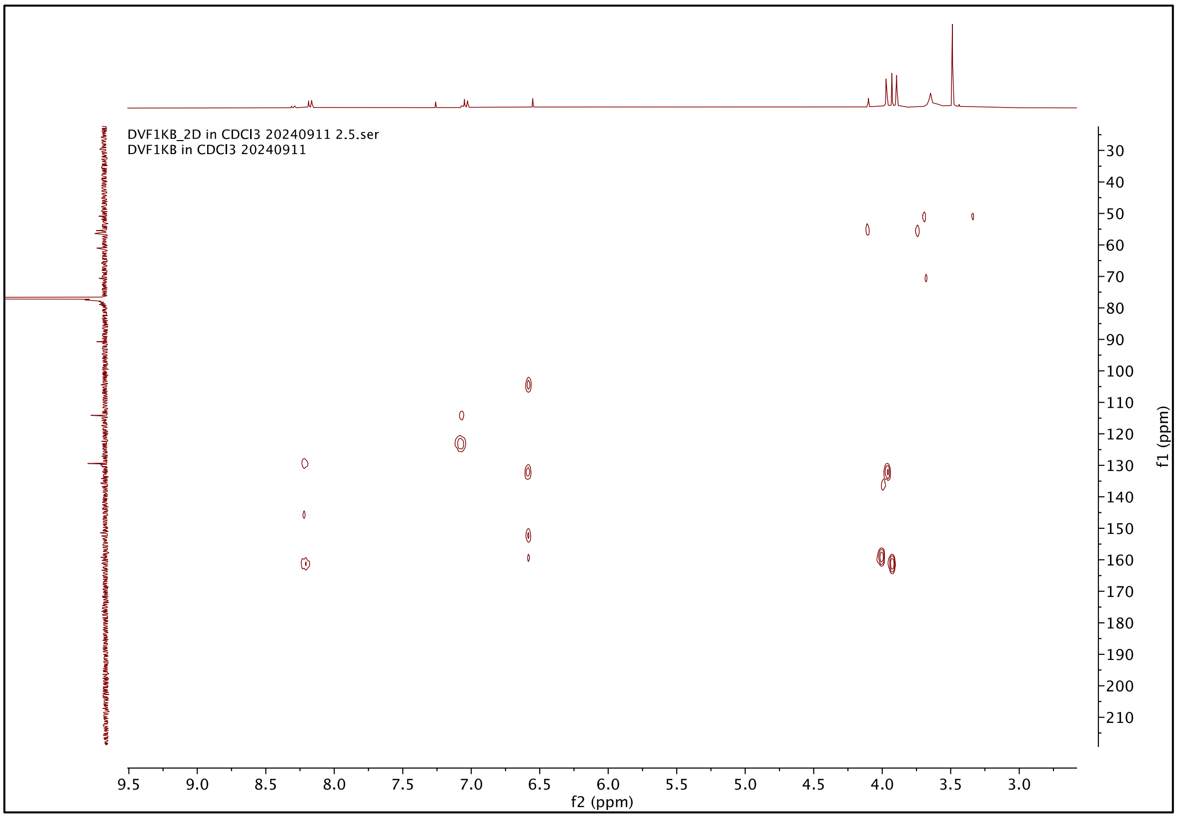

Figure S14. HMBC spectrum of compound **5**.

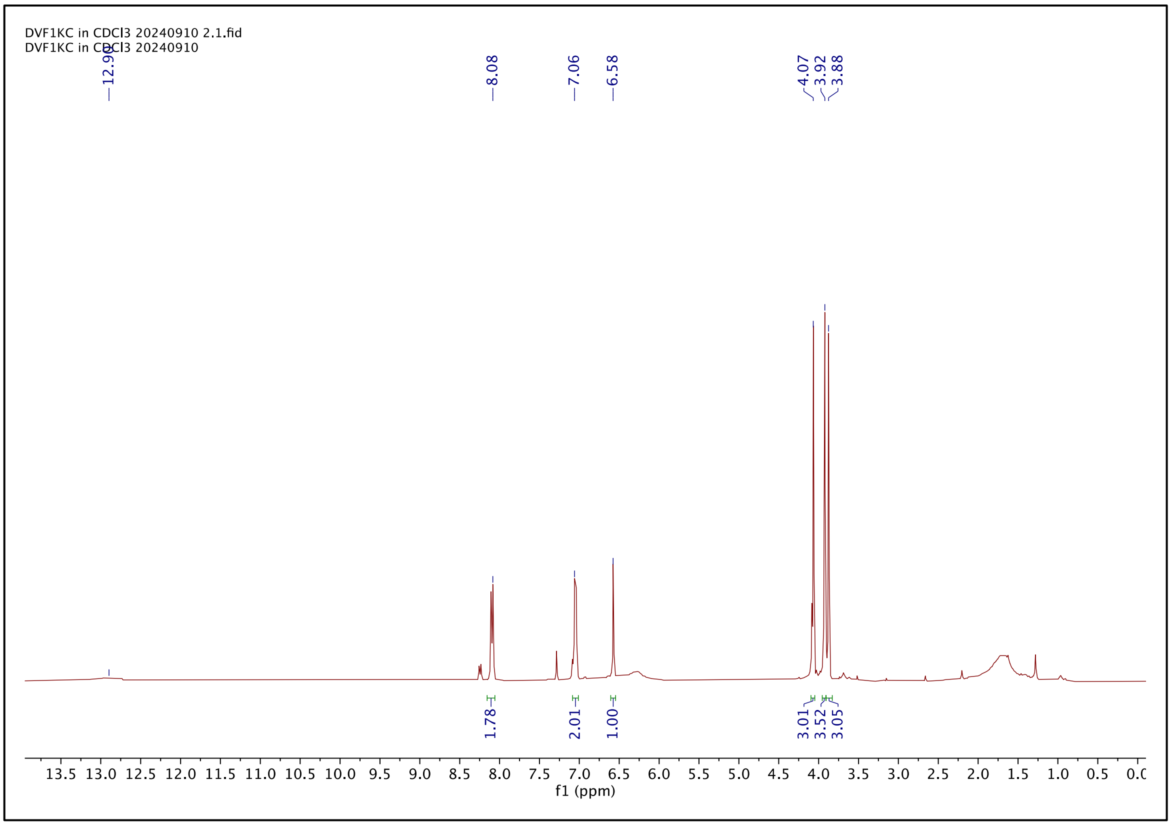

Figure S15. ^{1}H NMR spectrum (400 MHz, $CDCl_3$) of compound **6**.

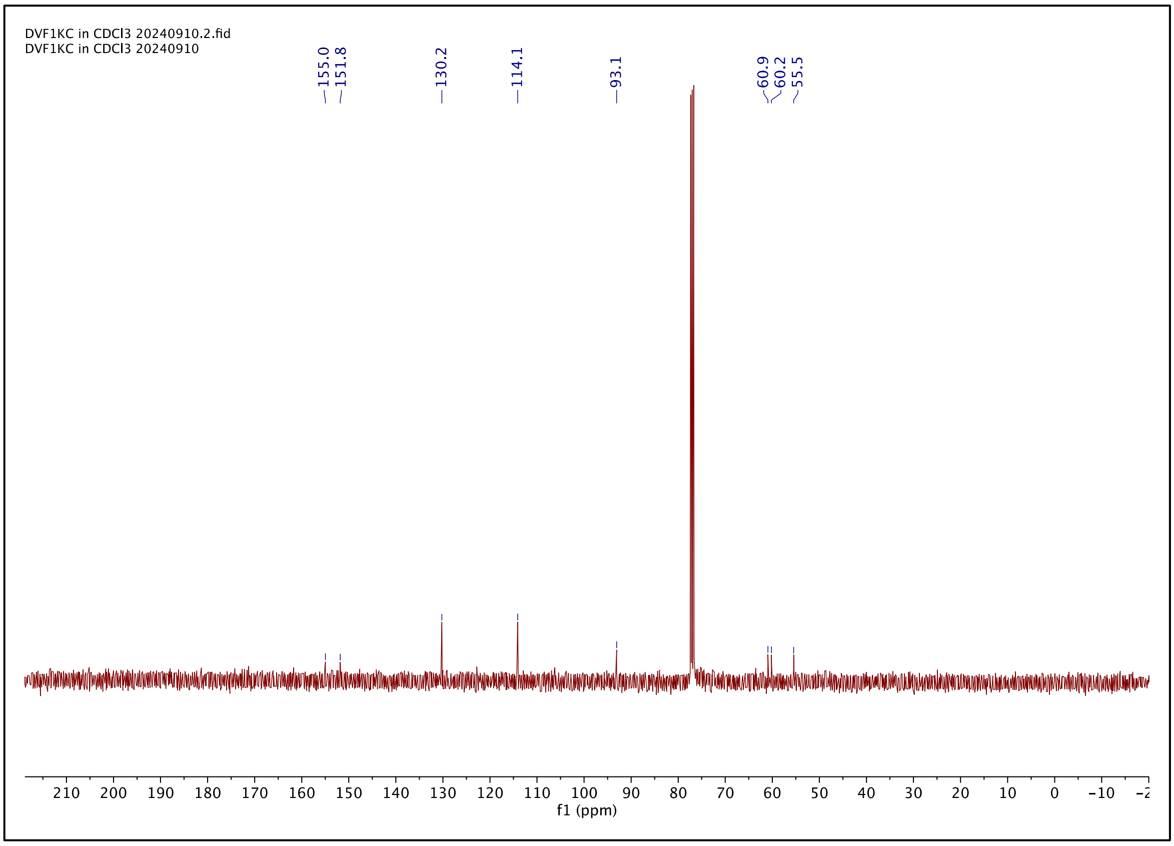

Figure S16. ^{13}C NMR spectrum (100 MHz, $CDCl_3$) of compound **6**.

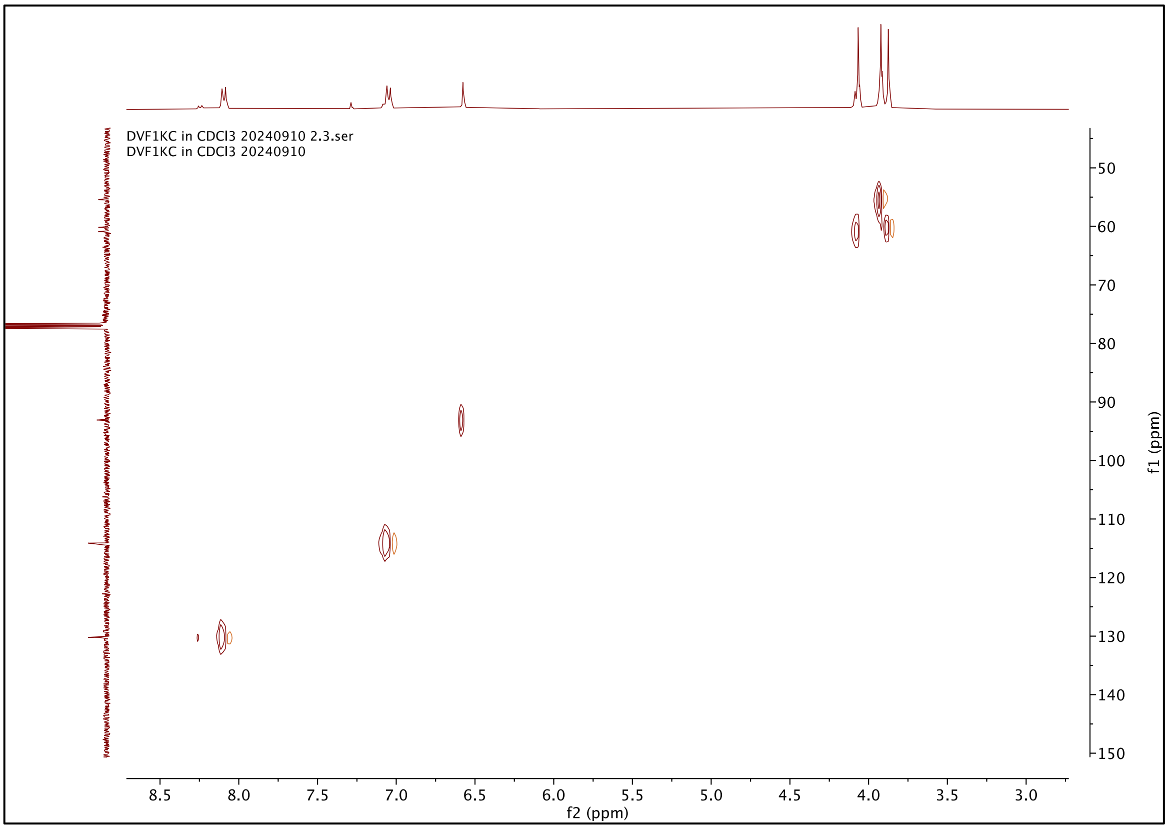

Figure S17. HSQC spectrum of compound **6**.

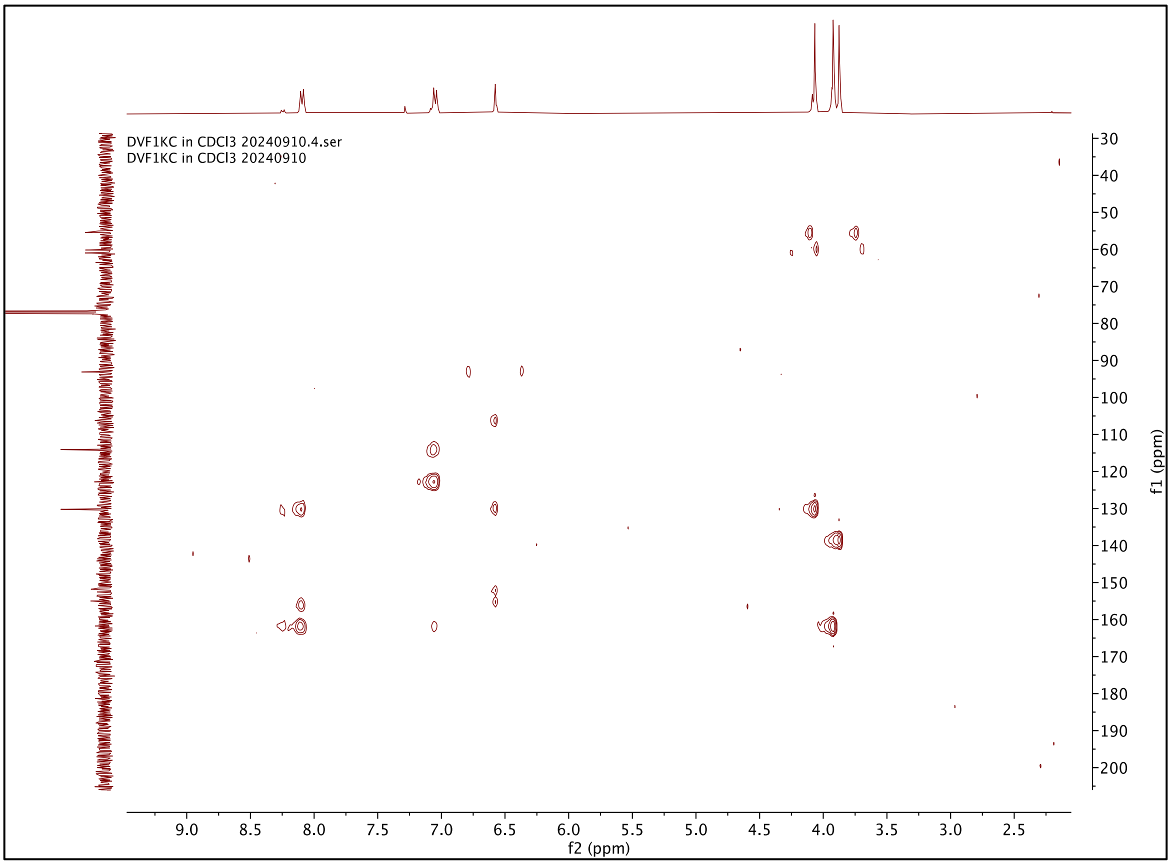

Figure S18. HMBC spectrum of compound **6**.

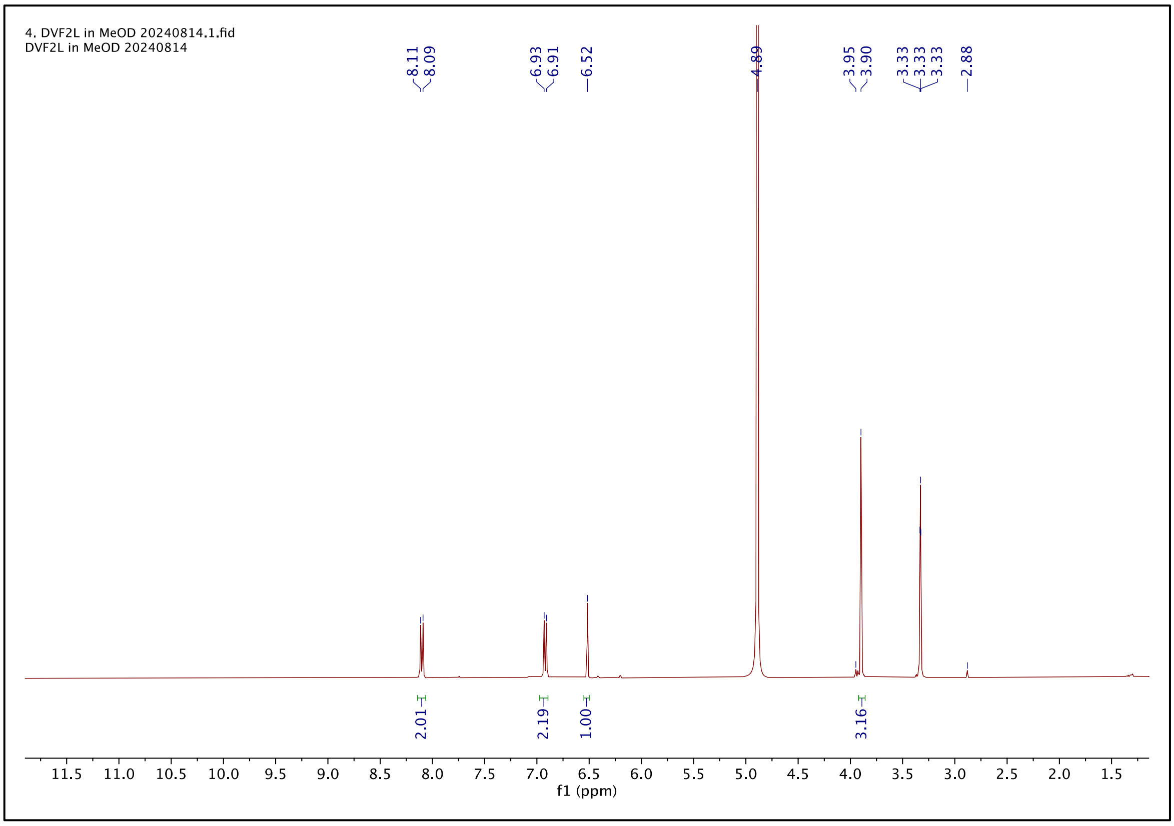

Figure S19. ^{1}H NMR spectrum (400 MHz, MeOD) of compound **7**.

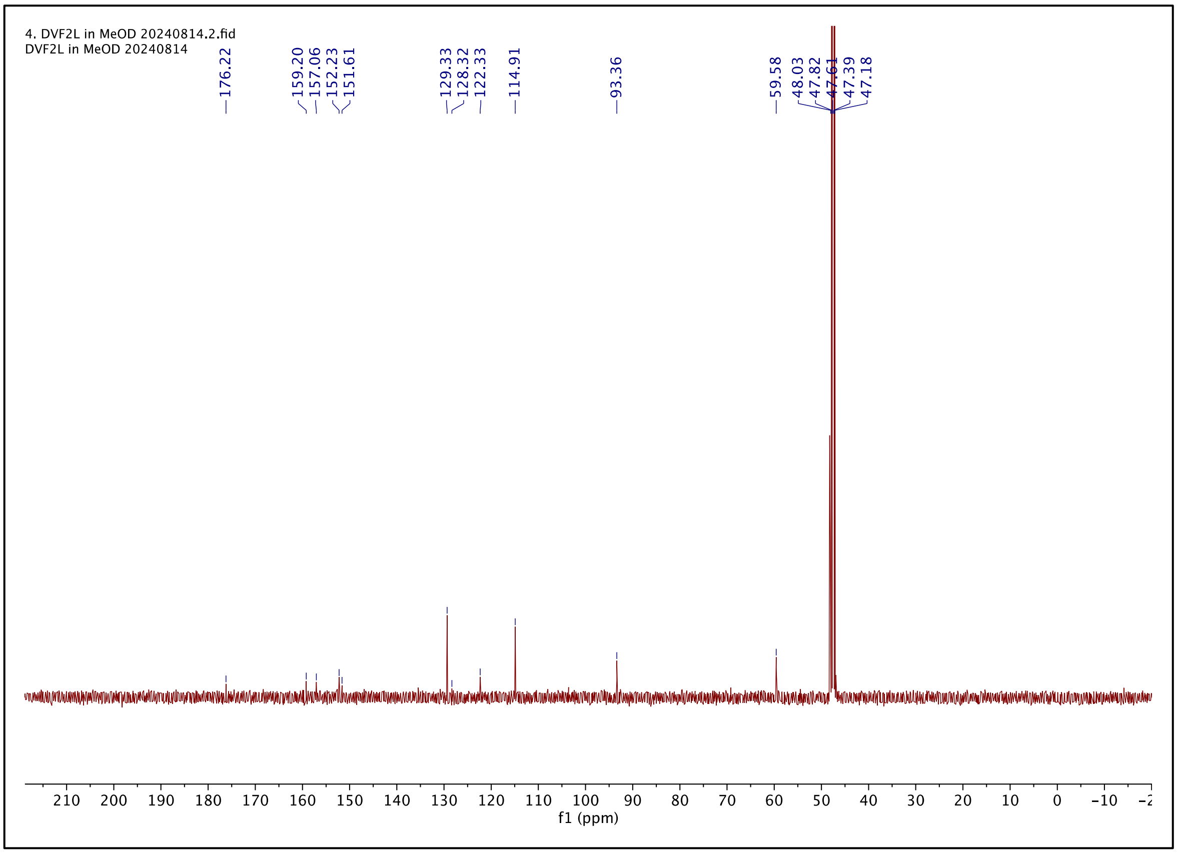

Figure S20. ^{13}C NMR spectrum (100 MHz, MeOD) of compound **7**.

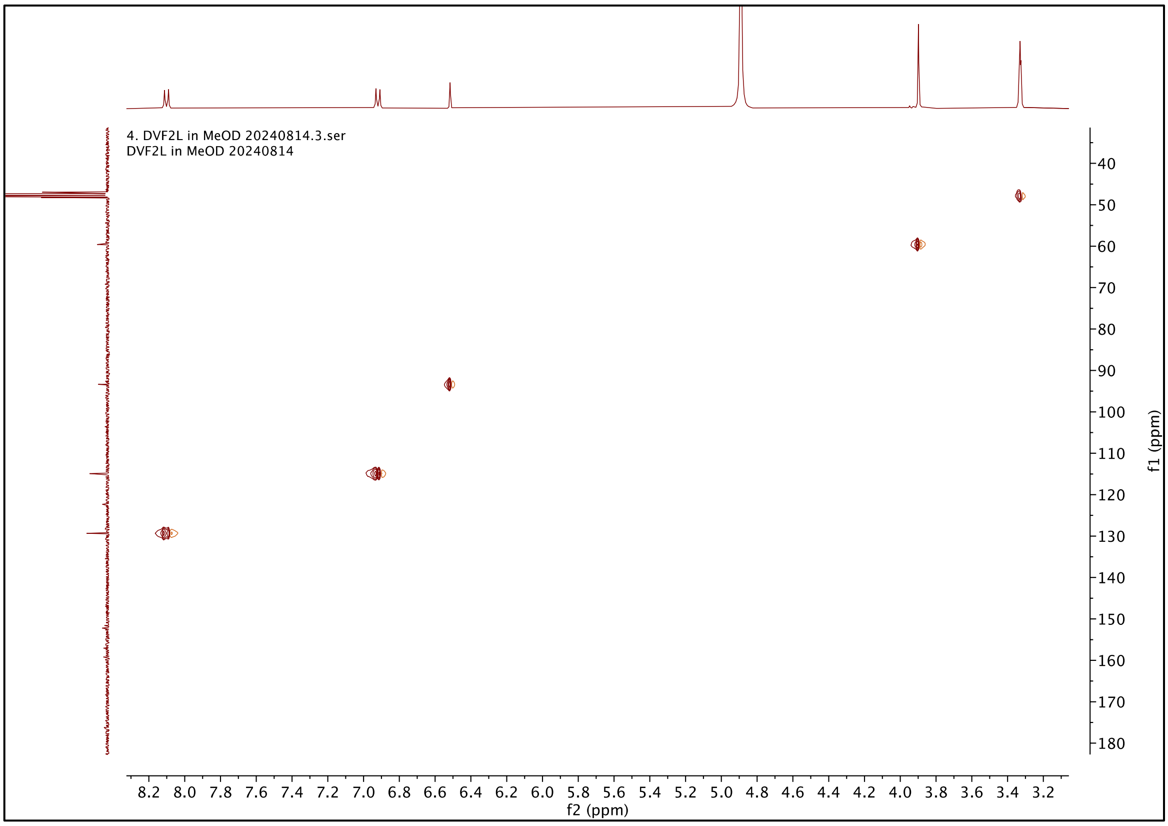

Figure S21. HSQC spectrum of compound **7**.

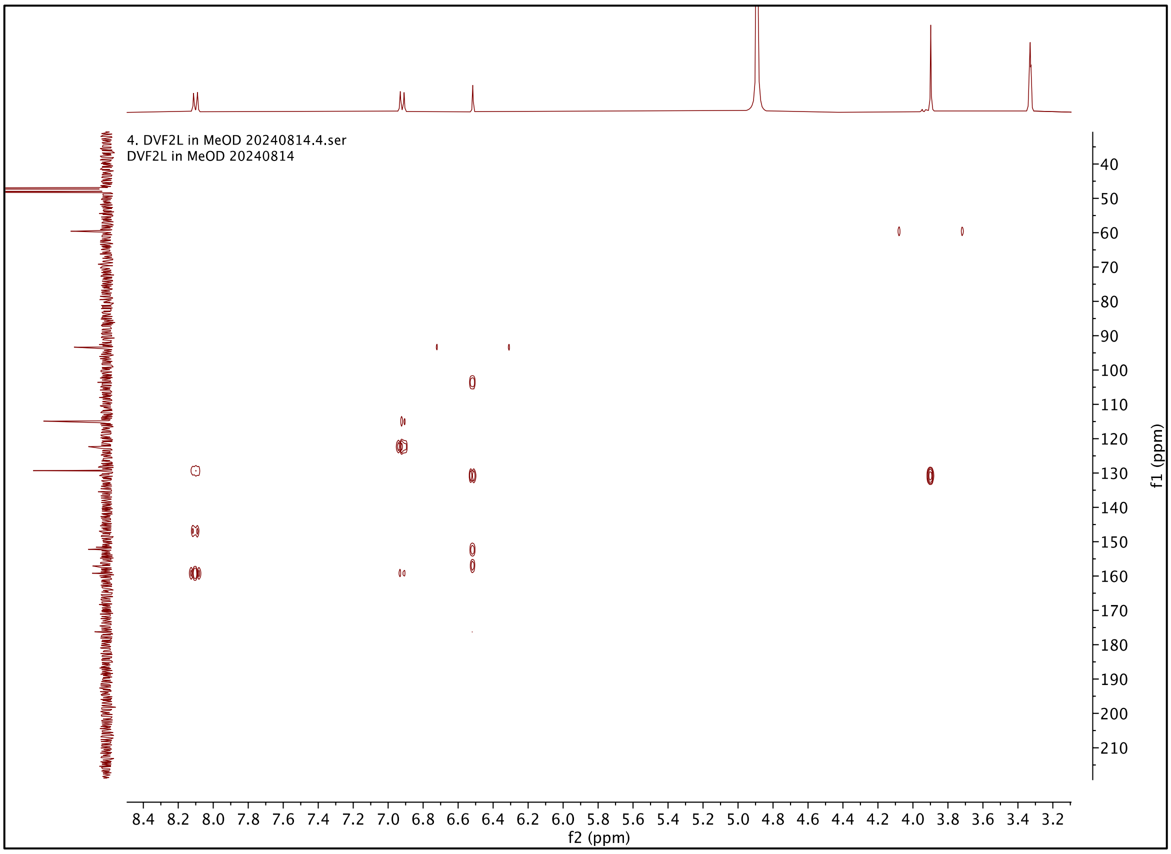

Figure S22. HMBC spectrum of compound **7**.

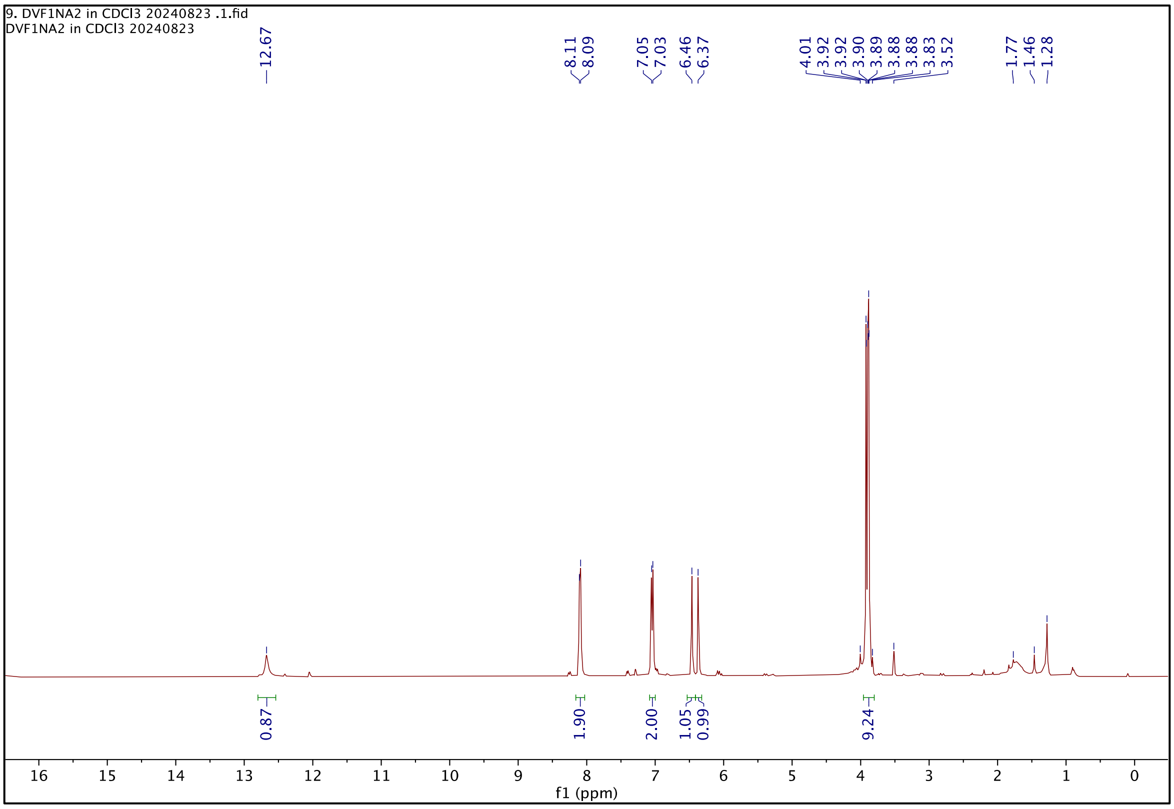

Figure S23. ^{1}H NMR spectrum (400 MHz, $CDCl_3$) of compound **8**.

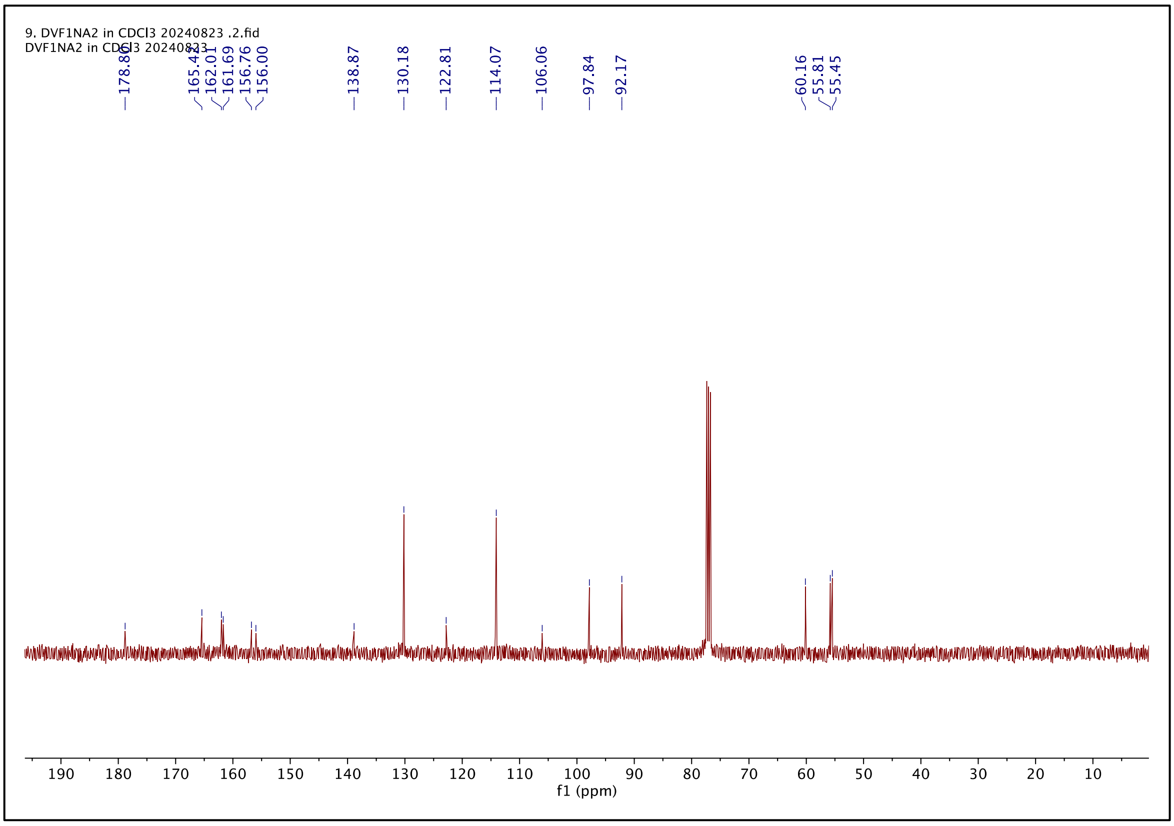

Figure S24. ^{13}C NMR spectrum (100 MHz, $CDCl_3$) of compound **8**.

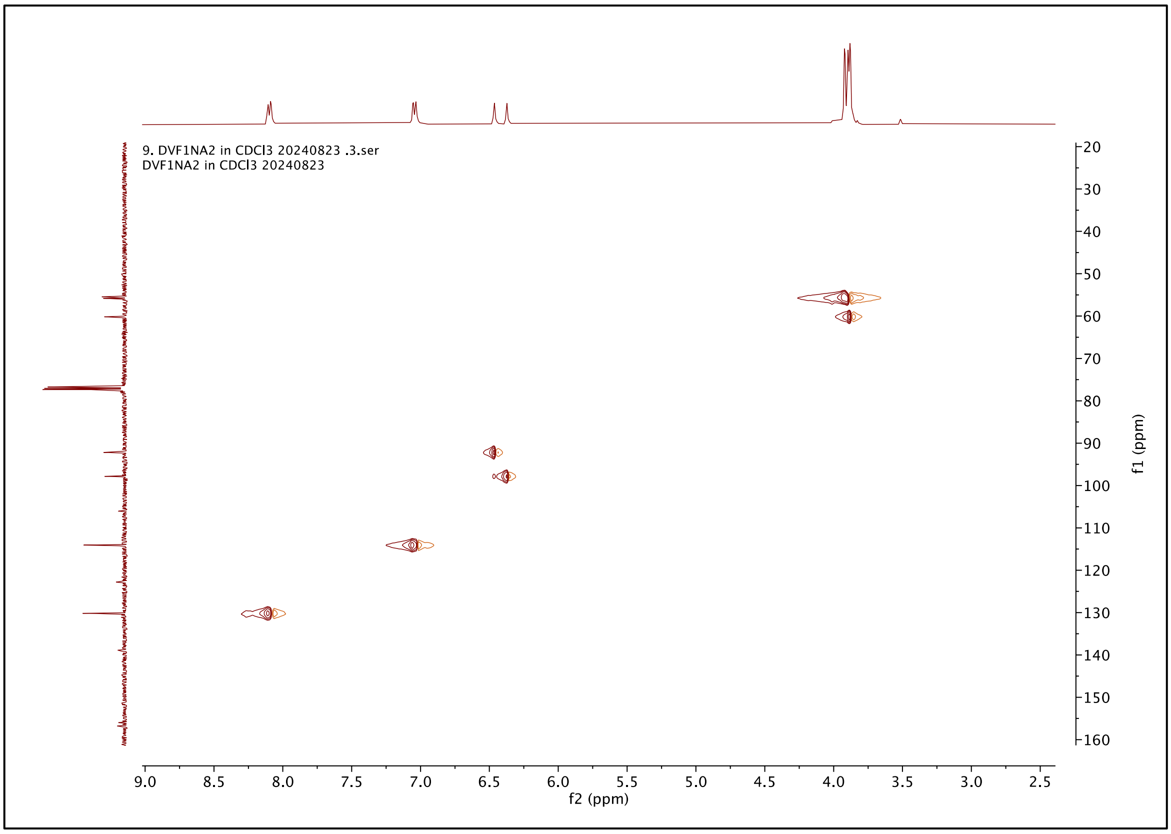

Figure S25. HSQC spectrum of compound **8**.

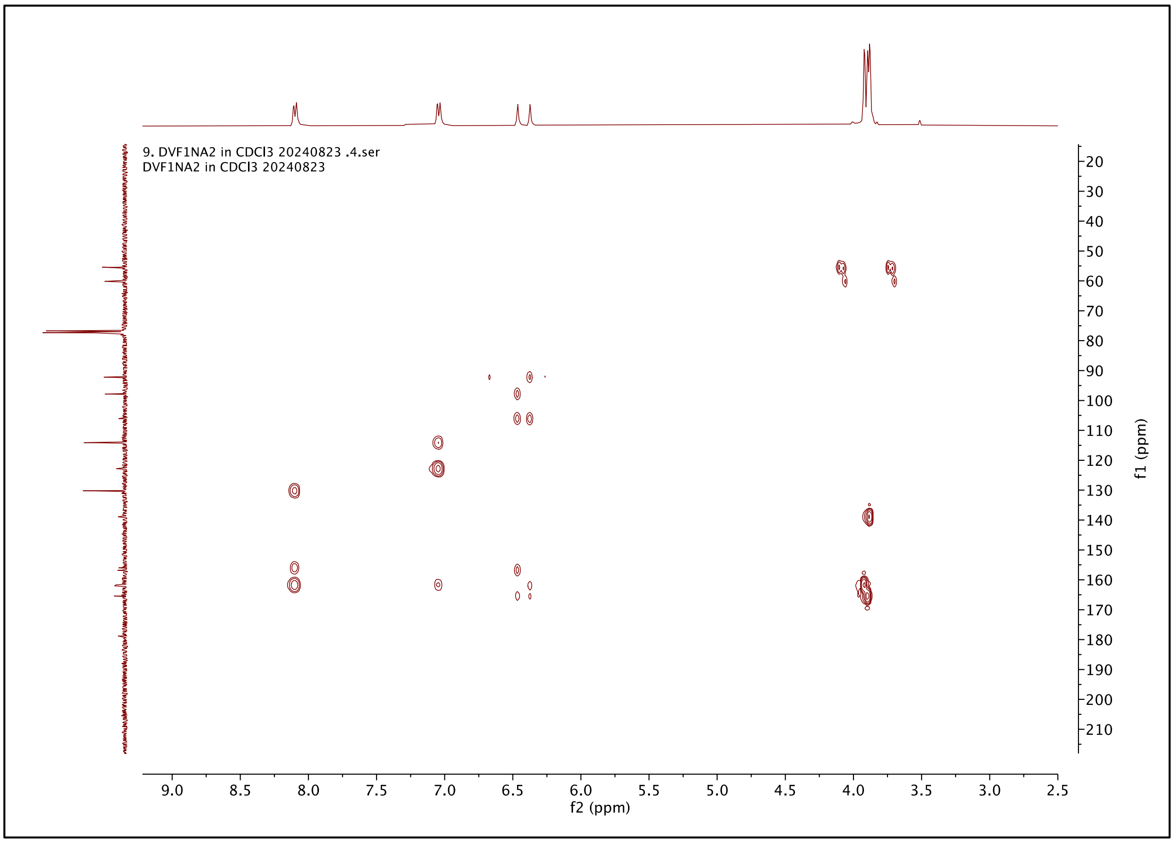

Figure S26. HMBC spectrum of compound **8**.

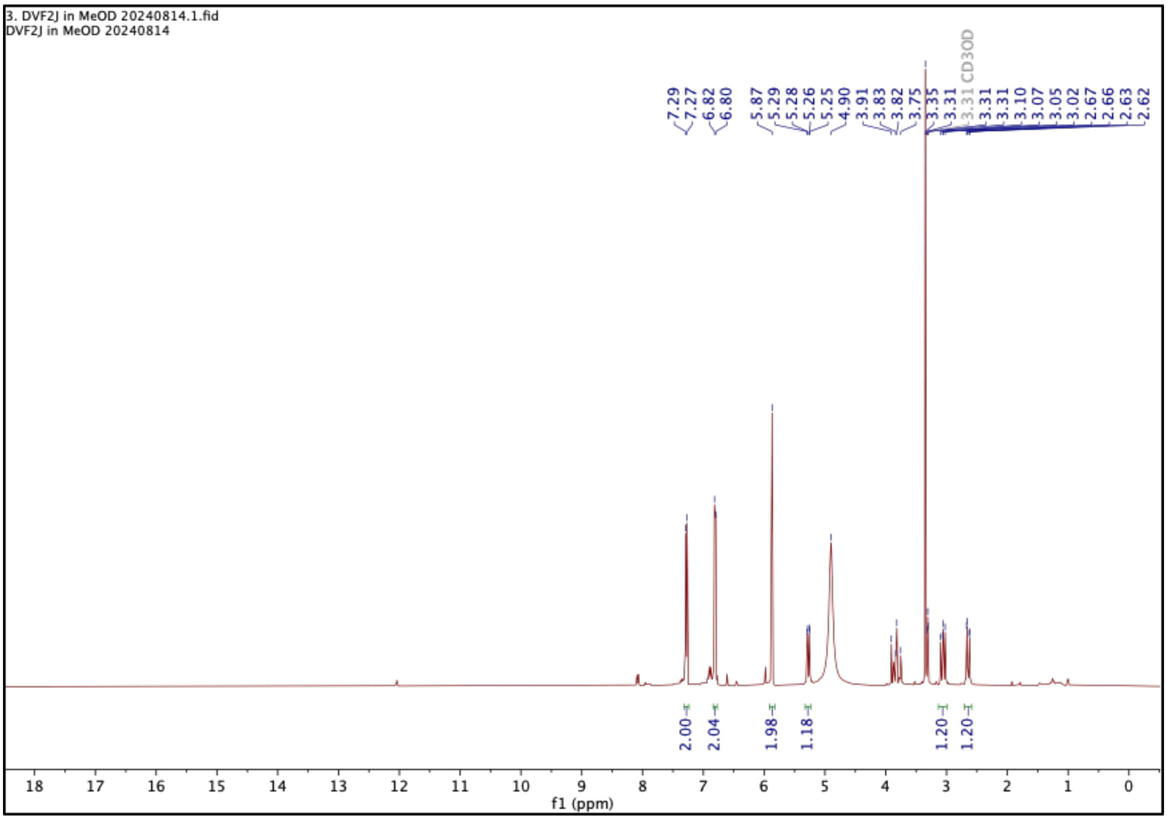

Figure S27. ^{1}H NMR spectrum (400 MHz, MeOD) of compound **9**.

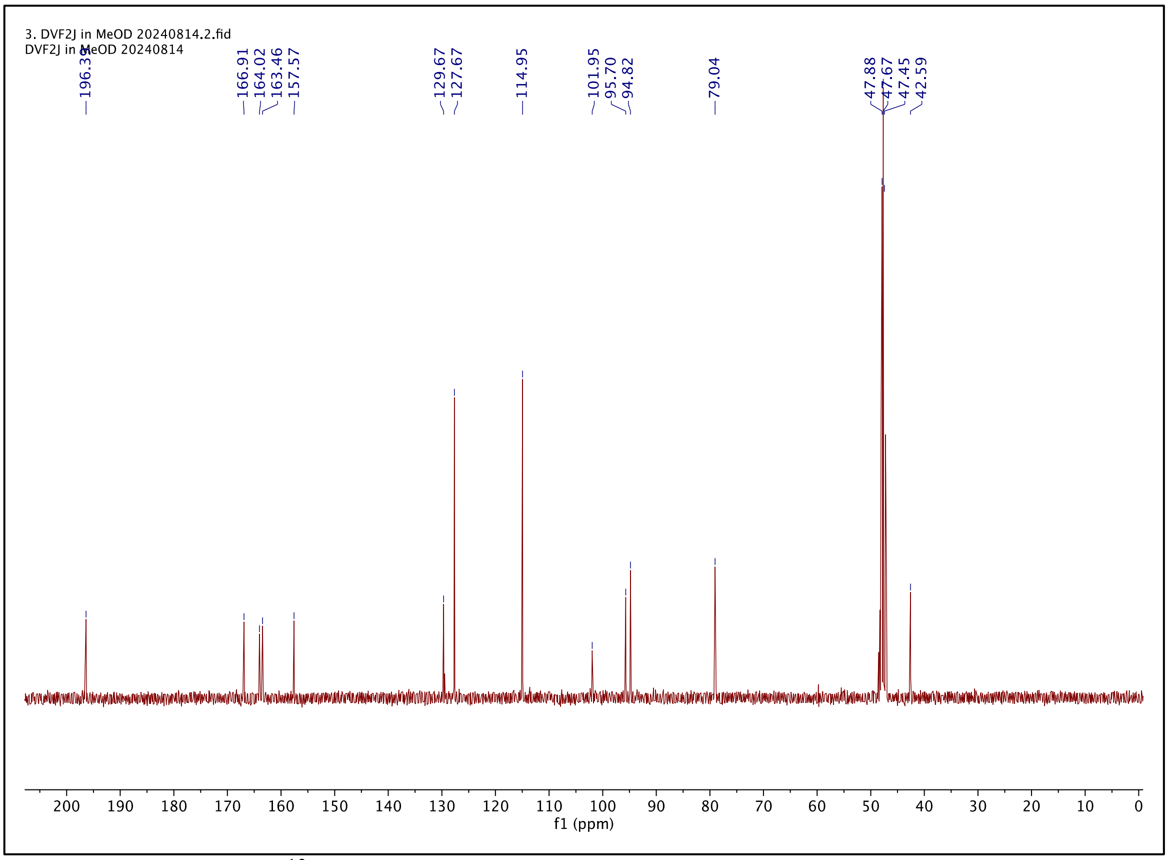

Figure S28. ^{13}C NMR spectrum (100 MHz, MeOD) of compound **9**.

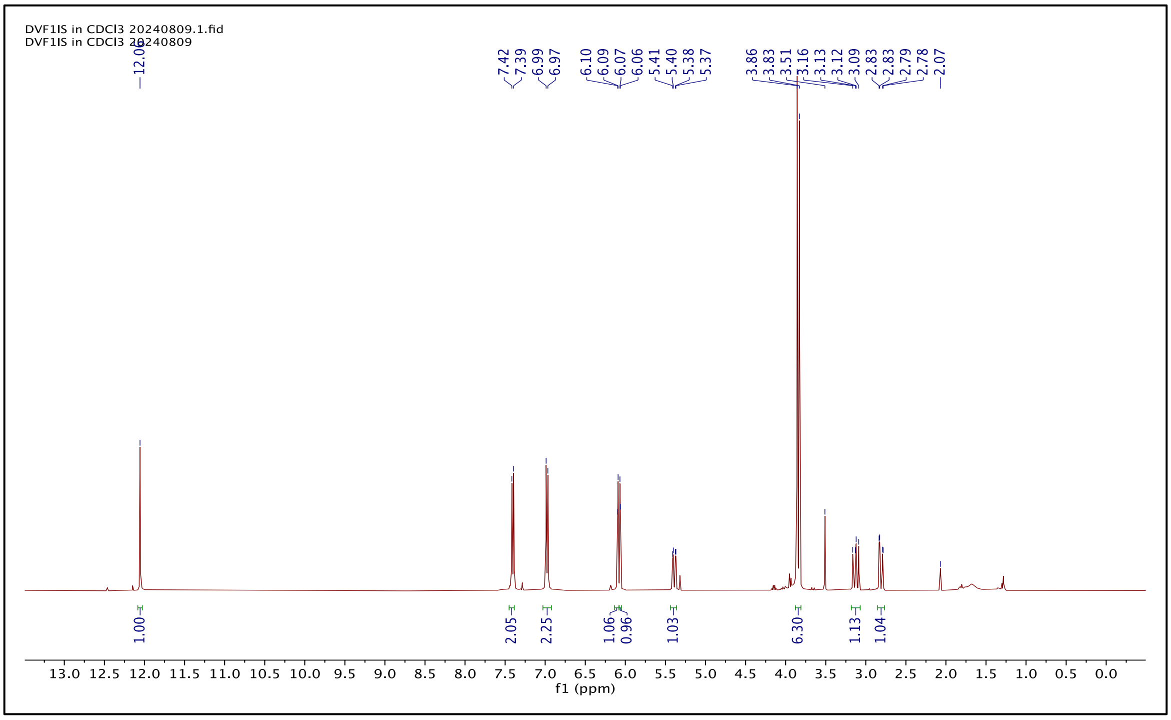

Figure S29. ^{1}H NMR spectrum (400 MHz, $CDCl_3$) of compound **10**.

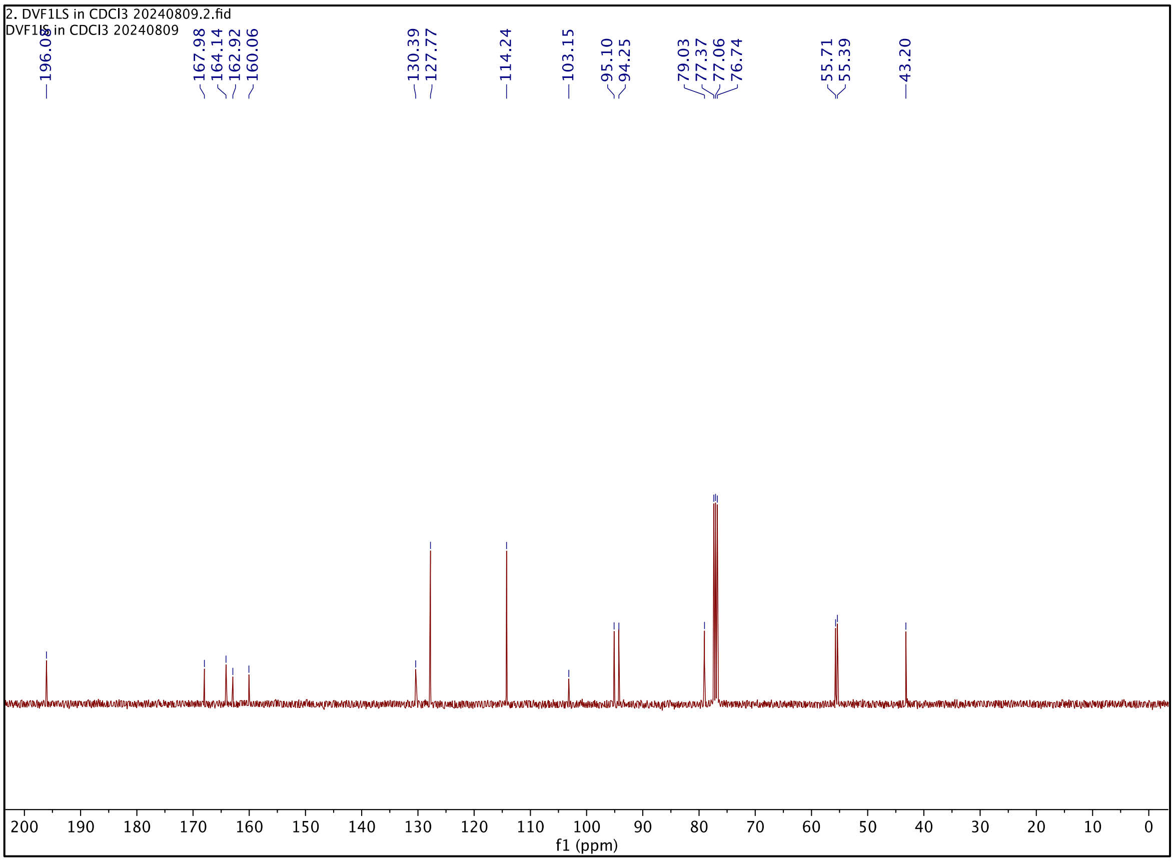

Figure S30. ^{13}C NMR spectrum (100 MHz, $CDCl_3$) of compound **10**.

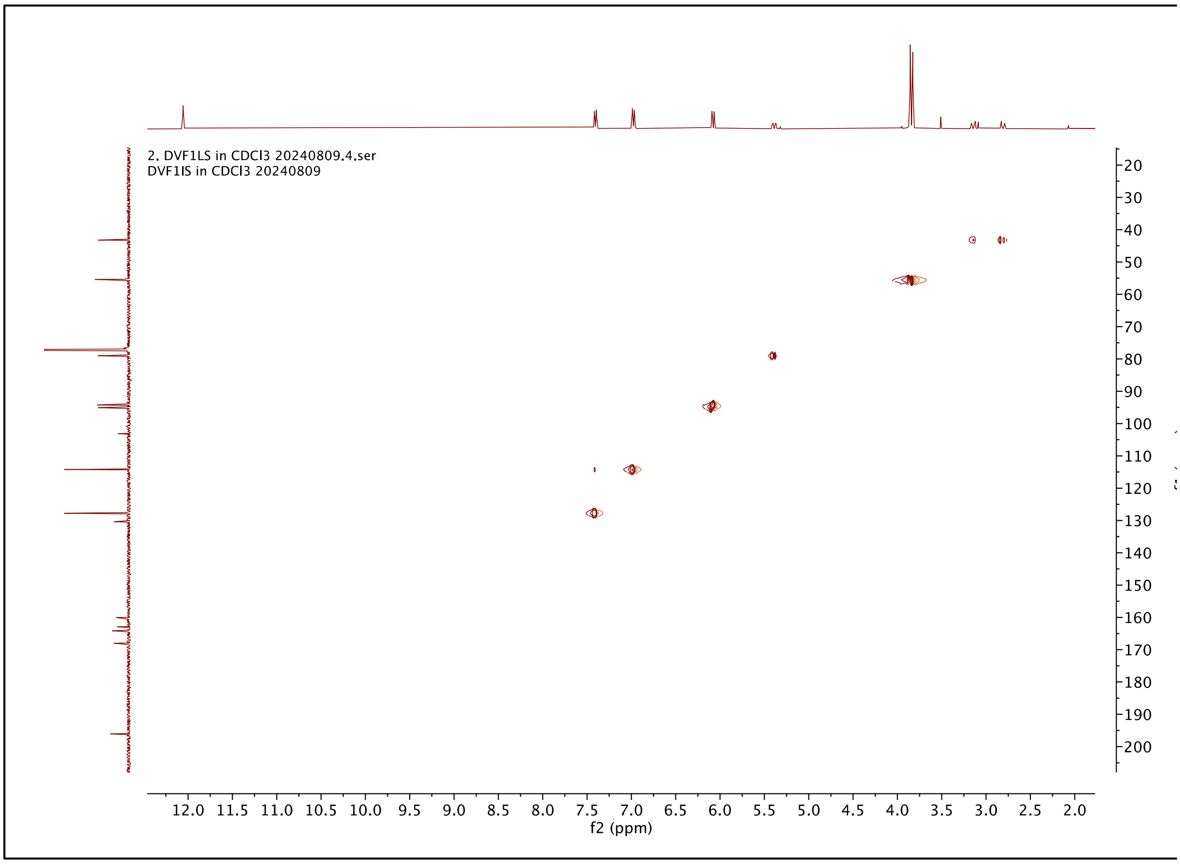

Figure S31. HSQC spectrum of compound **10**.

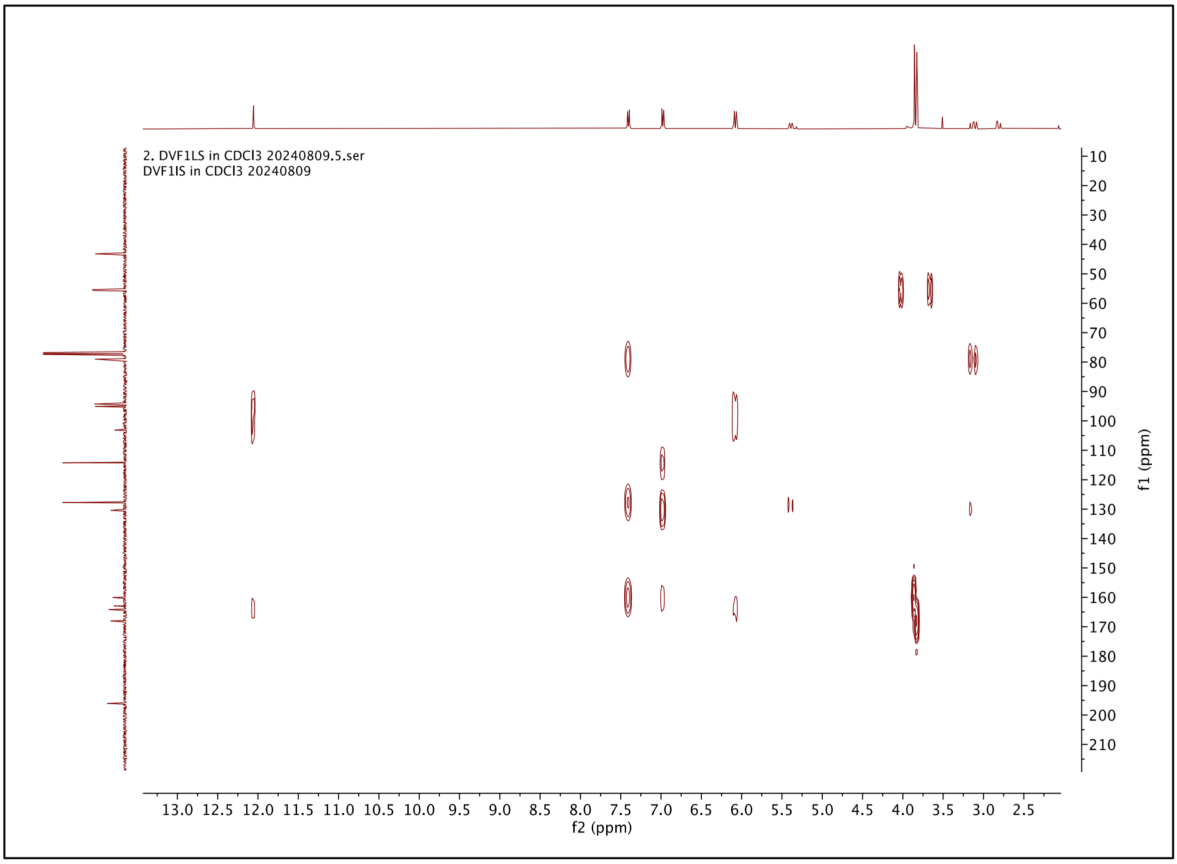

Figure S32. HMBC spectrum of compound **10**.

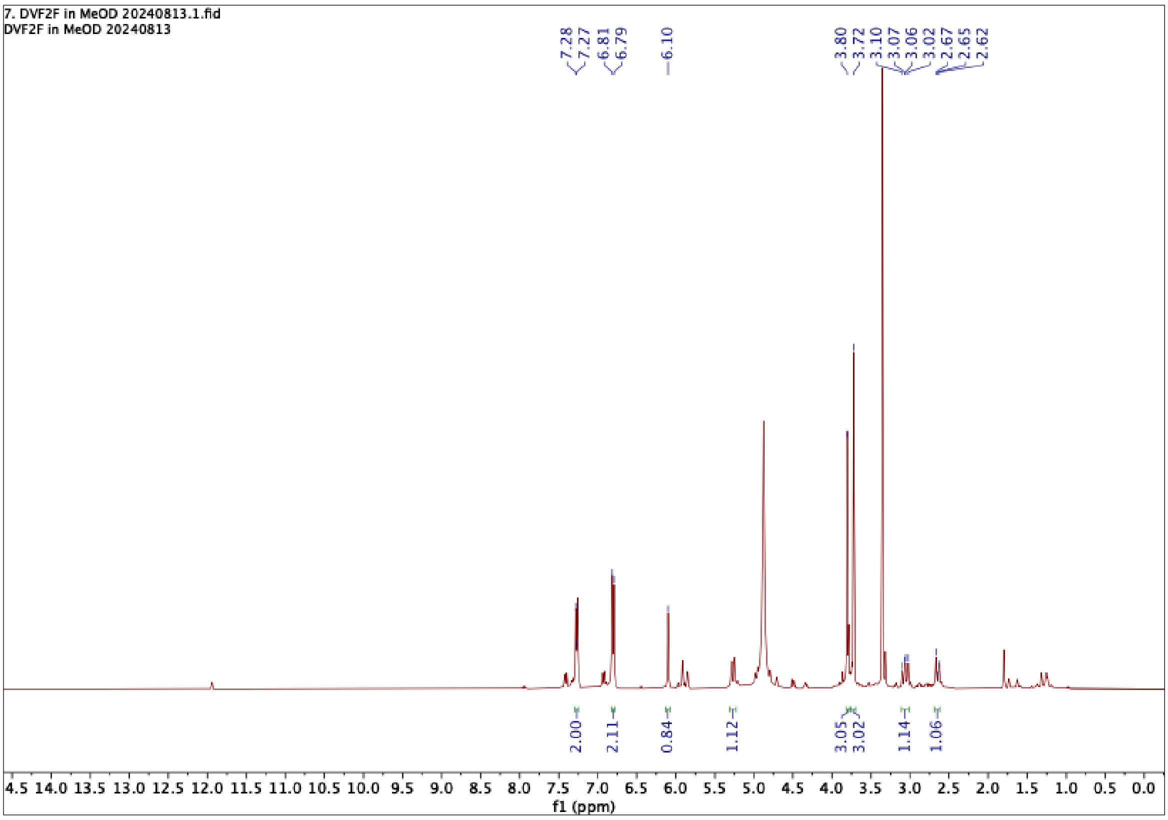

Figure S33. ^{1}H NMR spectrum (400 MHz, MeOD) of compound **11**.

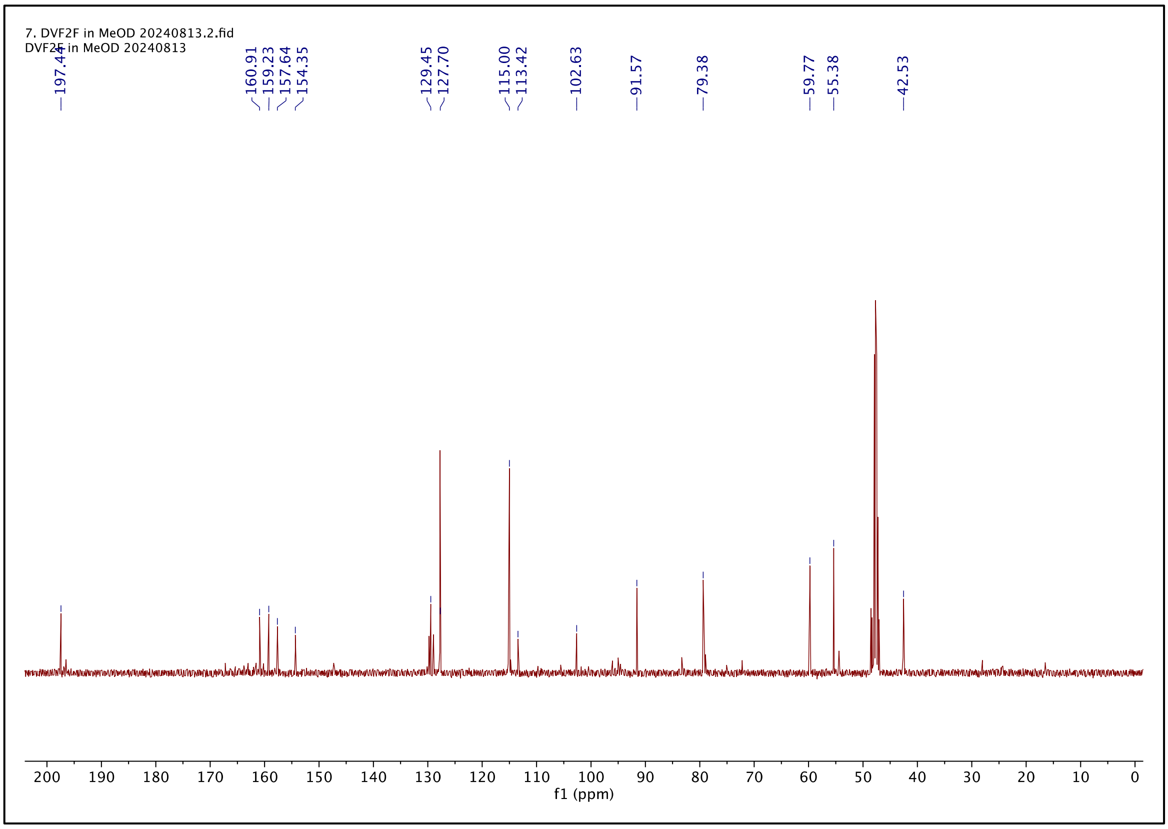

Figure S34. ^{13}C NMR spectrum (100 MHz, MeOD) of compound **11**.

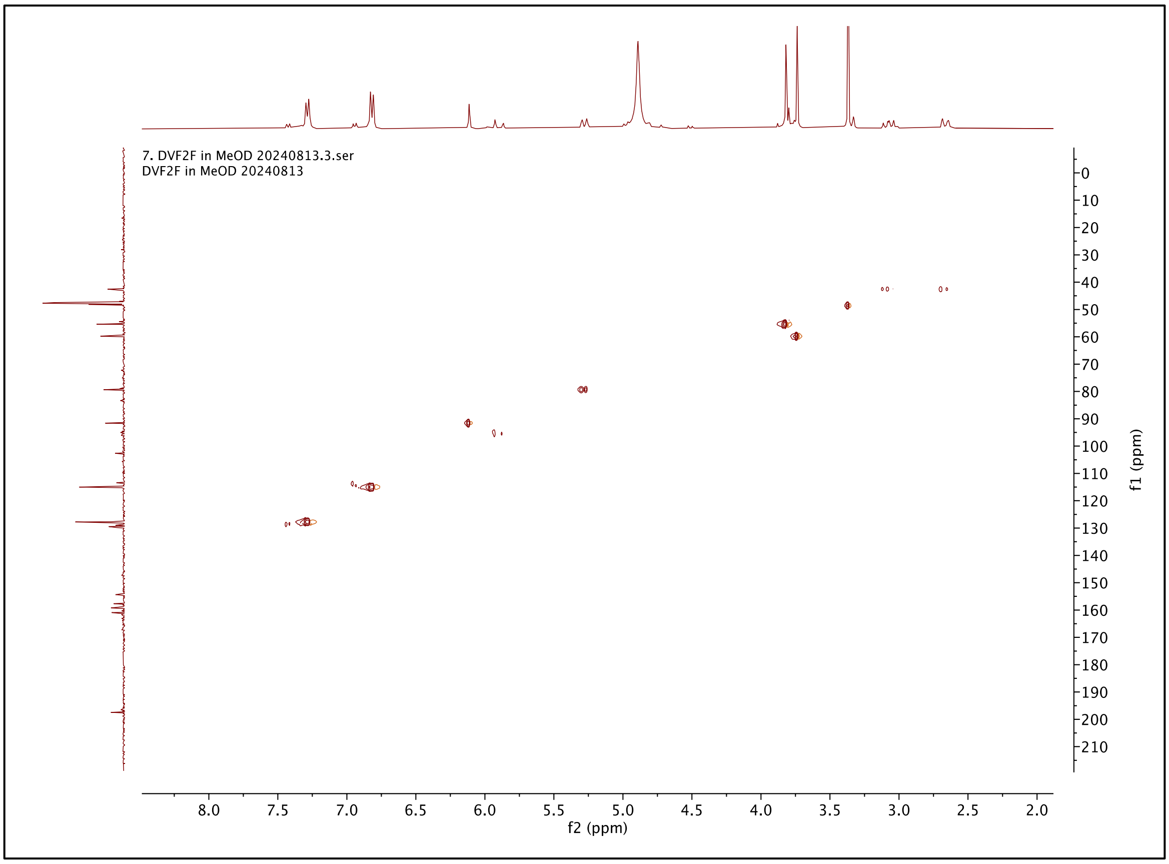

Figure S35. HSQC spectrum of compound **11**.

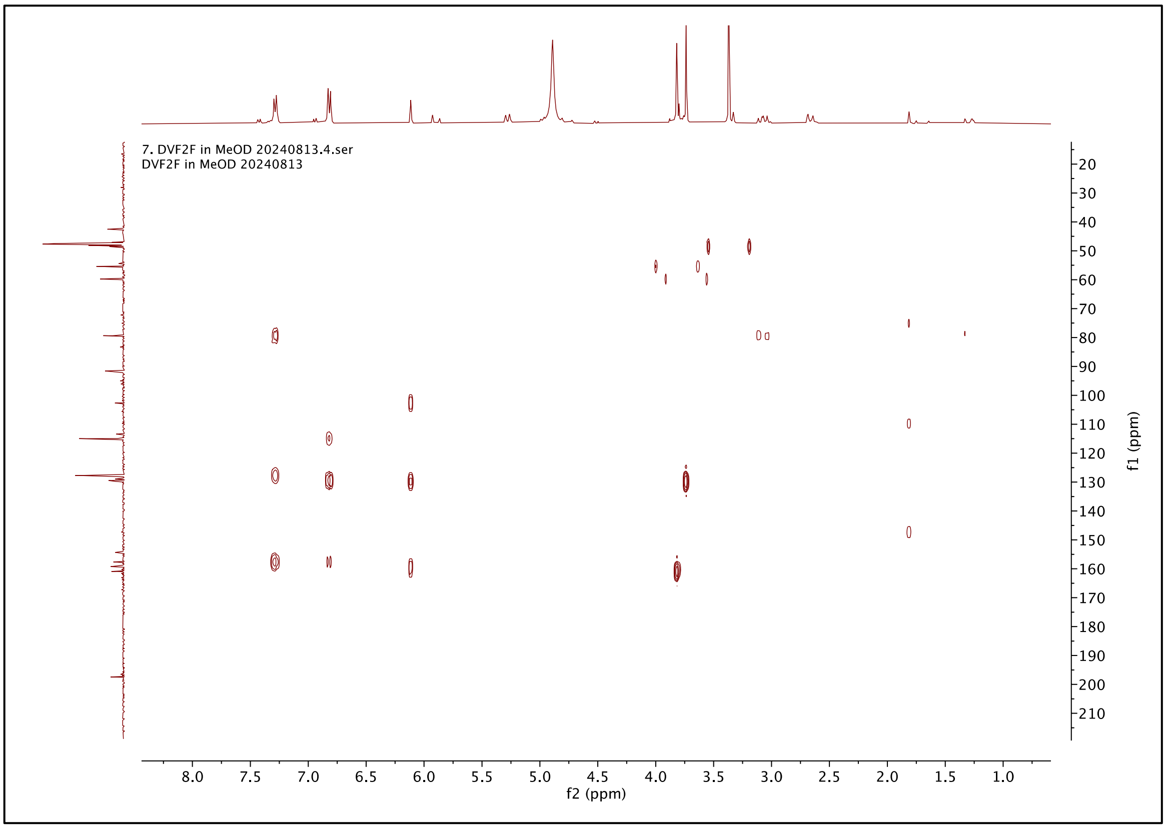

Figure S36. HMBC spectrum of compound **11**.

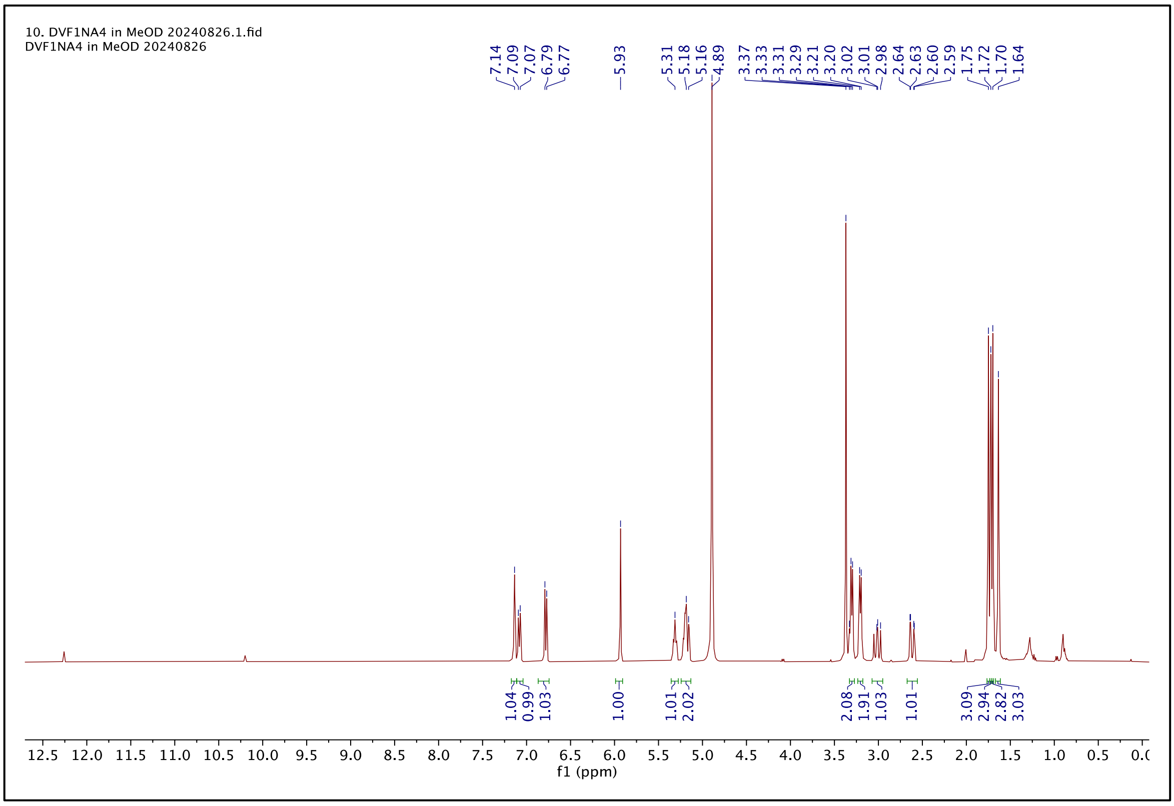

Figure S37. ^{1}H NMR spectrum (400 MHz, MeOD) of compound **12**.

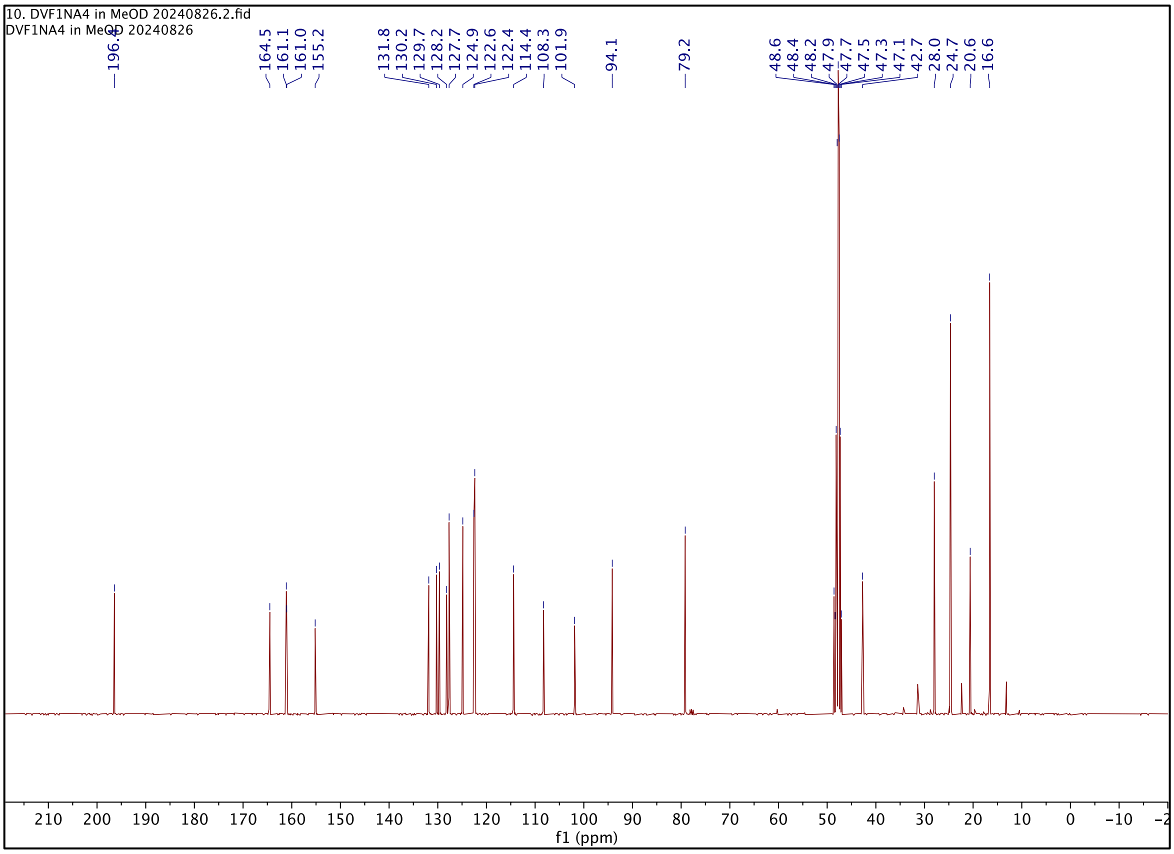

Figure S38. ^{13}C NMR spectrum (100 MHz, MeOD) of compound **12**.

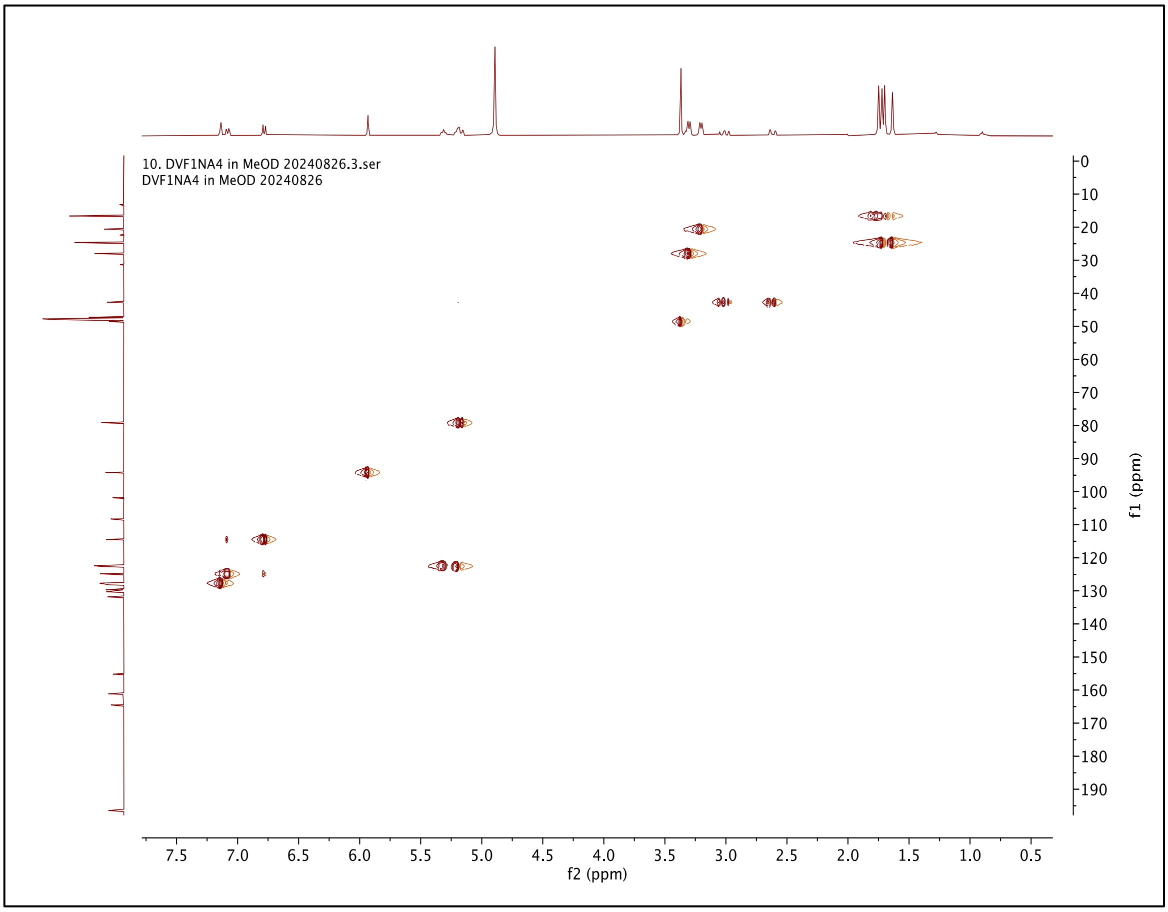

Figure S39. HSQC spectrum of compound **12**.

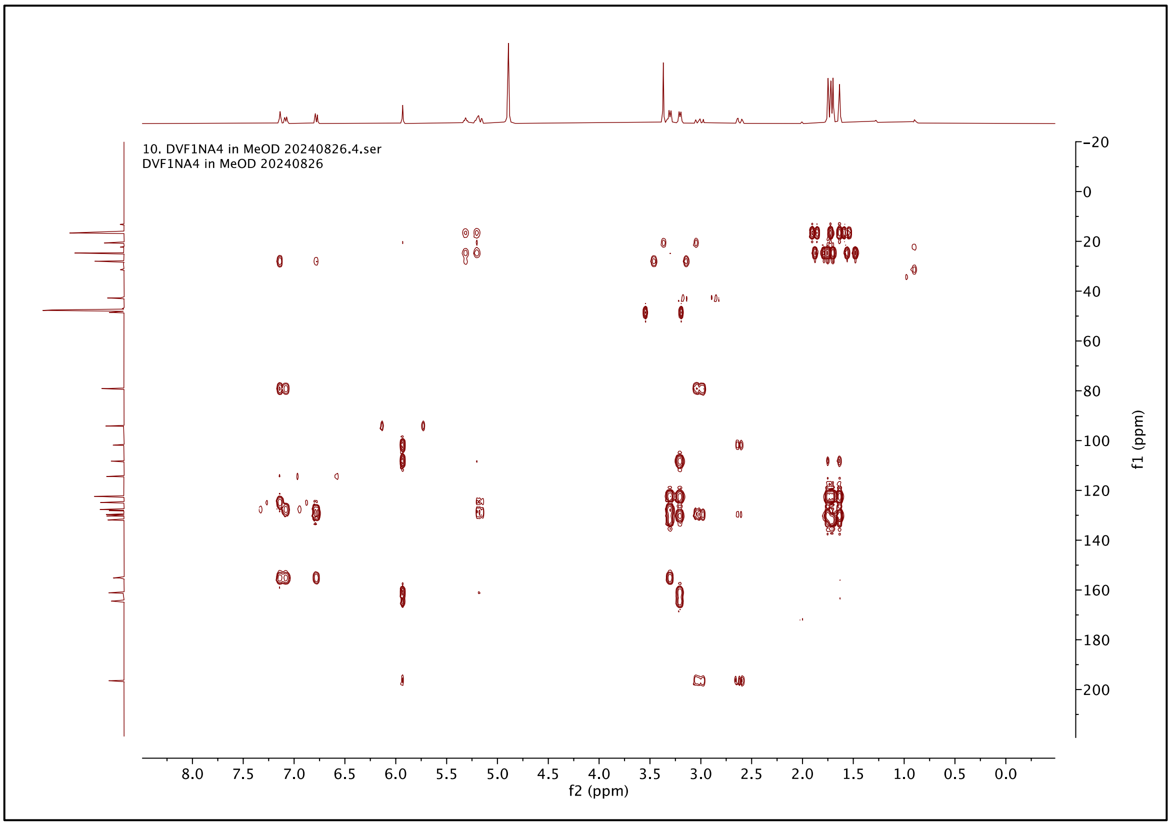

Figure S40. HMBC spectrum of compound **12**.

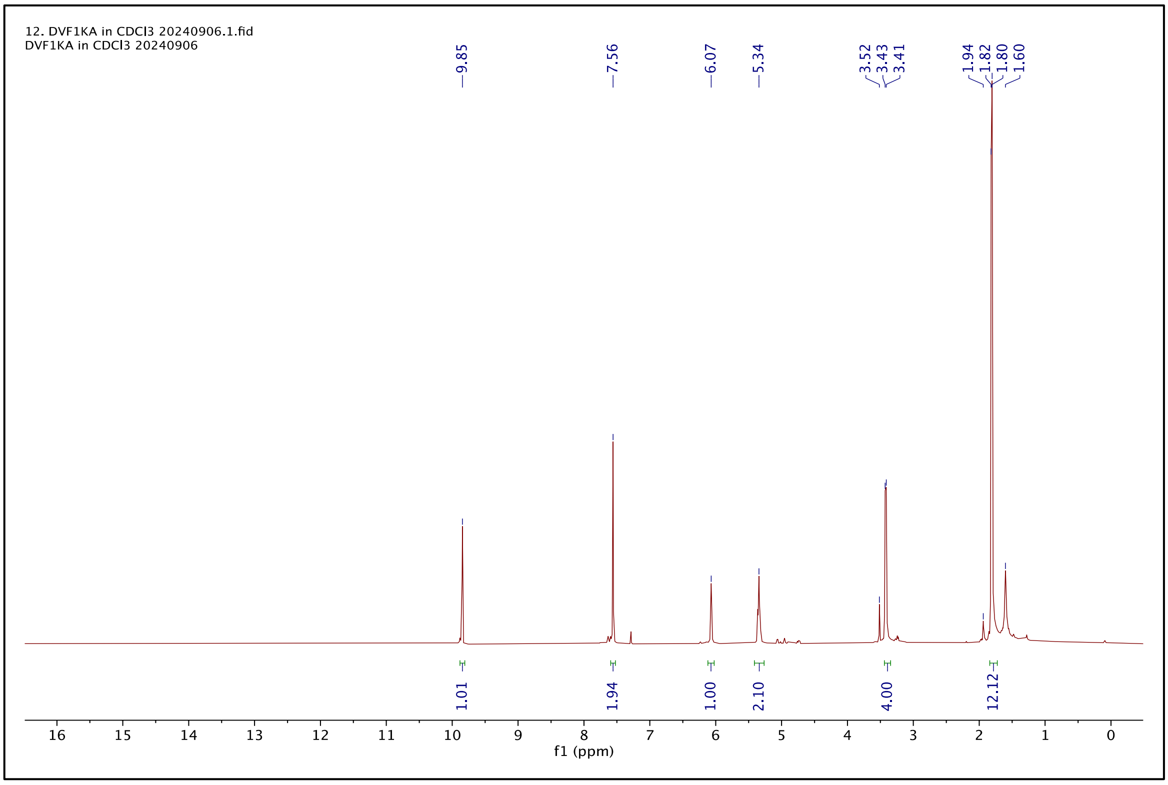

Figure S41. ^{1}H NMR spectrum (400 MHz, $CDCl_3$) of compound **13**.

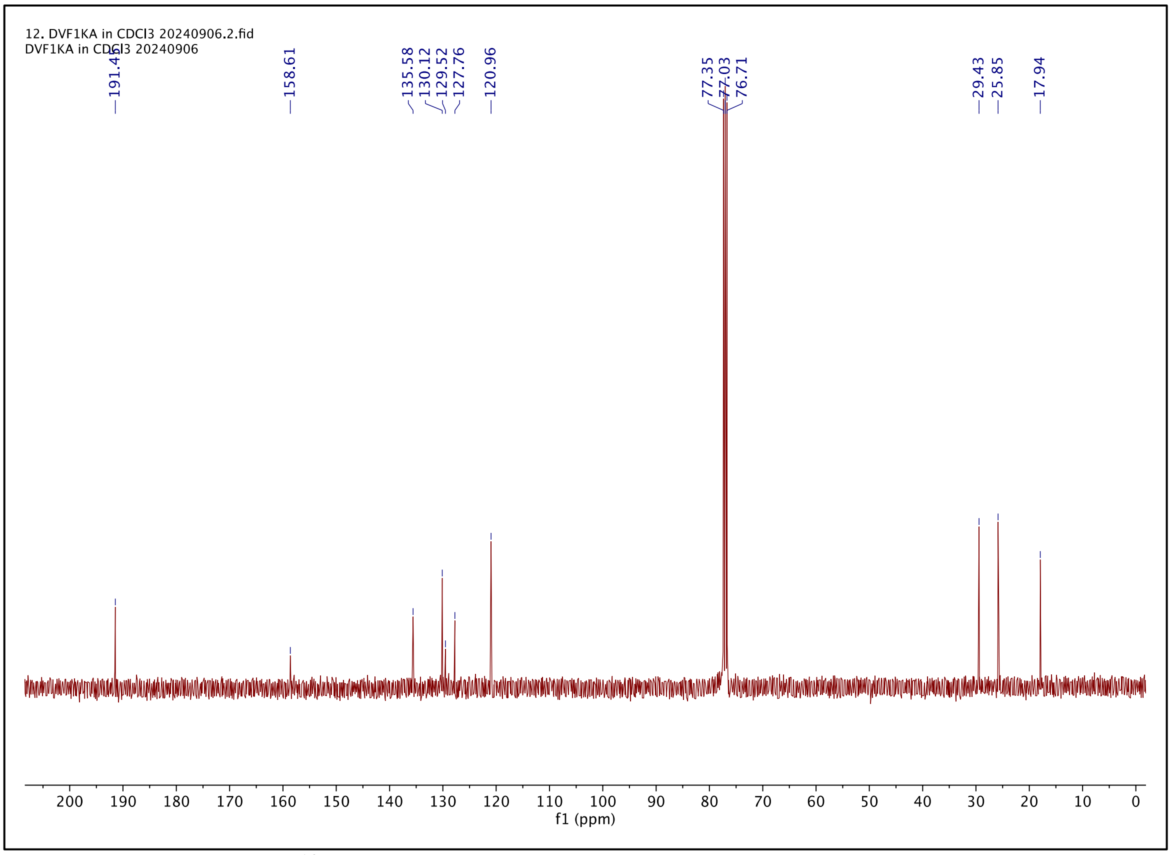

Figure S42. ^{13}C NMR spectrum (100 MHz, $CDCl_3$) of compound **13**.
